# Preparation and biomedical applications of Janus nanomotors

**DOI:** 10.1016/j.mtbio.2025.102444

**Published:** 2025-10-24

**Authors:** Banghui Wang, Tao Chen, Yixuan Li, Tong Yin, Zeyu Xi, Yuhan Guo, Yuanhong Xu, Xian-Ming Chu

**Affiliations:** a Department of Cardiology, The Affiliated Hospital of Qingdao University, Qingdao 266100, China; b Institute of Biomedical Engineering, College of Life Sciences, Qingdao University, Qingdao 266071, Shandong, China; c Qingdao University, Qingdao Medical College, Qingdao 266073, China

**Keywords:** Janus nanomotors, Preparation strategy, Targeted drug delivery, Precision biosensing, Minimally invasive therapeutics

## Abstract

Janus nanomotors (JNMs), as a forefront nanotechnology research focus, demonstrate great potential due to their special asymmetric structure and outstanding performance in disease diagnosis and treatment. These “two-faced” nanoparticles exhibit propulsion by leveraging asymmetric physicochemical properties, functioning as biological motors. In the biomedical field, they provide important applications, including targeted drug delivery, precision biosensing, and minimally invasive therapies, offering new directions and novel strategies for disease diagnosis and treatment. This paper reviews the current JNM fabrication methods and analyzes the advantages and limitations of each method as well as their respective advances in biomedical applications. Given the existence of multiple propulsion modes for JNM, they can be categorized into three types based on the quantitative characteristics of their power sources: externally field-driven JNMs, fuel-driven JNMs, and composite-driven JNMs. Based on the distinct origins of driving forces, externally field-driven JNMs are further categorized into light-driven, magnetically-driven, ultrasound-driven, and electric-driven types. Fuel-driven JNMs are classified into enzyme-driven and self-driven variants. Furthermore, this paper specifically highlights composite-driven JNMs, which achieve functional enhancement in complex biological environments by integrating multiple stimulus sources. Finally, we discuss future prospects and challenges for differently propelled JNMs, anticipating their significant influence on human health.

## Graphical abstract

**Figure:**
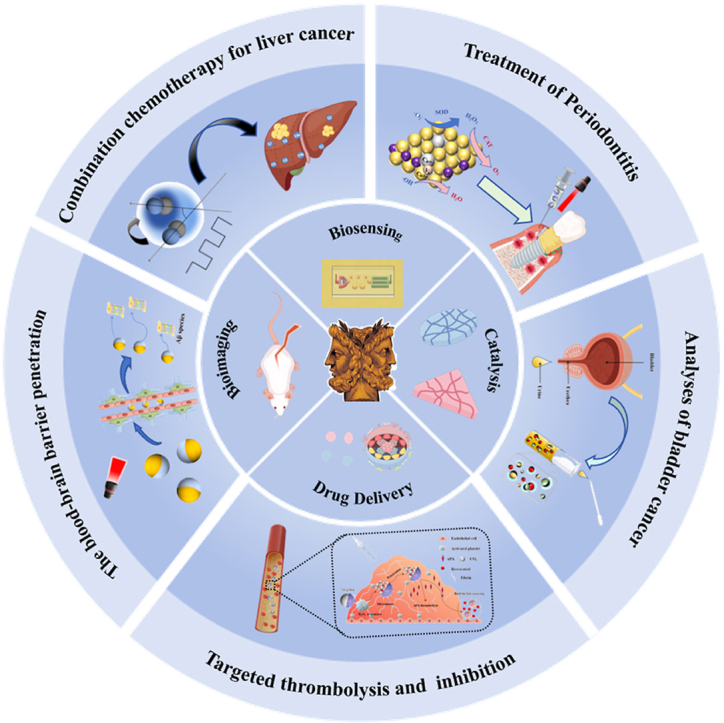

## 1 Introduction

With the rapid development of nanotechnology, nanomotors have attracted much attention due to their breakthrough applications in targeted delivery, imaging, biocatalysis, and other fields. It can convert different forms of energy into mechanical energy and demonstrate excellent motion performance in various environments [1]. Among them, Janus nanomotors (JNMs) have become a cutting-edge hotspot of nanomotors by virtue of its unique asymmetric structure and controllable motion mechanism [2]. Janus originated from the Roman double-faced god, and the Janus material concept was first introduced by French scientist Pierre in his 1991 Nobel lecture [3,4]. His research team designed a glass sphere with two hemispheres, polar and non-polar, an asymmetric structure that could enable the nanomotor to have both physical and chemical properties [5,6]. Most traditional nanomotors have symmetrical structures and are driven by uniform chemical gradients and external fields to achieve functions such as penetrating biological barriers, targeted drug delivery, and penetrating tumors [7,8]. In contrast, JNMs has two or more distinct regions on both sides of the nanomotor and possesses different chemical and physical properties, making it anisotropic in composition and surface characteristics and enhancing its versatility [9,10]. This anisotropy allows JNMs to have a variety of properties such as environmental adaptation, multi-field response, efficient motion, and multi-functional integration. Compared with the single drug delivery function of traditional nanomotors, JNMs are able to achieve a variety of applications, such as targeted drug delivery, imaging, sensing, *etc.*, with these unique properties, breaking the limitation of applications [[11], [12], [13]]. In short, due to the asymmetry of its structure, the Janus nanomotor can build modules with different functions on both sides of a single nanoparticle (NP), independently integrating the “drive module” and the “treatment/diagnosis” module to meet the requirements of multiple functions. Meanwhile, JNMs, relying on their asymmetric structure, can adapt to two or more driving modes, enhancing biological adaptability.

JNMs has been widely studied by a large number of researchers due to its excellent performance and can be prepared by methods such as phase separation, self-assembly, and masking [5]. In recent years, the research on JNMs has made rapid progress. Researchers have adopted various driving methods to better adapt it to different biological application environments [[14], [15], [16], [17]]. Externally field-driven JNMs can be categorized into light-driven, magnetically-driven, ultrasound-driven, and electric-driven types. Among them, light-driven JNMs can enhance tissue penetration by utilizing the near-infrared (NIR) photothermal effect and can be applied in drug delivery, thrombolysis, and antibiotics [18,19]. The use of magnetic fields allows precise control of the direction of motion and targeting of JNMs, reduces side effects, and modulates the rate of drug release [20]. Similar to magnetoactuation, the movement of JNMs can also be driven by using ultrasound, and ultrasound-driven JNMs are widely used in liquid environments, in addition to releasing drugs under ultrasound stimulation and prolonging the half-life of drugs through intelligent response carriers [[21], [22], [23]]. Electric-driven JNMs rely on the effect of an electric field and can significantly improve tissue targeting and penetration [24,25]. Fuel-driven JNMs can include enzyme-driven JNMs and self-driven JNMs. Enzyme-driven JNMs utilize *in vivo* biofuels (*e.g.*, hydrogen peroxide, glucose) to generate gas or concentration gradient-driven motions through catalytic decomposition [[26], [27], [28]]. Self-driven JNMs are a more unique type of JNMs. It is based on the chemical reactions of internal components and achieves movement through the reaction force of gases. It has a wider range of applications and multiple advantages, such as targeting and penetration [29,30]. In addition to the JNMs driven by a single driving force as described above, multiple driving modes can also be combined for driving, integrating the advantages of various driving modes to enhance the efficiency and biological adaptability of the nanomotor [31].

Currently, traditional biomedical diagnostic and therapeutic means are facing serious challenges in terms of target delivery efficiency, lesion penetration depth, therapeutic controllability, and reduction of side effects. To overcome the barriers of complex biological environments and achieve precise and efficient personalized treatments, there is an urgent need to develop new intelligence-driven therapeutic platforms, and the emergence of JNMs has opened up promising new directions and strategies to break through these bottlenecks. The core innovation of JNMs lies in the unique asymmetric structure that converts the environmental energy or its own characteristics into the ability to move autonomously, thus realizing the active targeting in the biological medium, The high flexibility and breadth of biomedical applications of JNMs mark a key step towards the intelligentization of nanomedicine, providing abundant potential for high-precision diagnosis and treatment of diseases in the future. It is hoped that the different preparation methods of JNMs and the specific applications of JNMs with different driving modes in biomedical applications introduced in this review will stimulate the readers' thinking and provide new possibilities for the development of JNMs.

## 2 Preparation of JNMs

JNMs have a non-centrosymmetric structure, consisting of an asymmetric distribution of at least two different chemistries, functional groups, or polar regions in a variety of shapes. This unique asymmetric structure can endow the JNMs with diverse functional properties. Some JNMs are amphiphilic and can reduce the interfacial tension between oil and water, which makes them excellent in emulsion stabilization and oil-water separation. Meanwhile, JNMs can be integrated with multiple functions, and different regions can be loaded with substances with different functions. In therapeutic applications, JNMs enable the synergy of chemotherapy and photothermal therapy, enhancing treatment efficacy. Regarding bio-imaging, the integration of magnetic and fluorescent properties in JNMs allows for dual-modal MRI and fluorescence imaging. These capabilities endow JNMs with notable advantages across multiple research and clinical fields, as supported by previous studies [7,32]. In the following, we will discuss in detail the three major synthesis strategies for JNMs: phase separation, self-assembly, and masking, and analyze the advantages and disadvantages of each (Table 1) [7].

**Table 1:** Preparation strategy and characterization of JNMs.

Strategy		Shape of JNMs	Principle	Advantage	Disadvantage	Application scenarios	Ref.
Phase separation	**Microfluidic**	1) Dumbbell-like 2) Snowman-shaped 3) Eccentric spherical	The solvent is dissolved and evaporated by adjusting the temperature	Size-controlled	1) Relatively wide particle size distribution 2) Lower productivity	Not clearly corresponding to a single driving type, mainly used for preparing JNMs with uniform size and controllable shape (such as dumbbell shaped and snowman shaped)	[7,20,32,[53], [54], [55]]
	**Seed-mediated polymerization**	1) Shell-like 2) Snowman-shaped	Nuclei were prepared by emulsion polymerization and then shells were loaded onto the surface of the nuclei	1) Simple and controlled 2) Suitable for polymer assembly	1) Precise control of monomer proportions 2) Harsh conditions	1) Light-driven 2) Magnetically-driven	[7,32,53,[56], [57], [58], [59]]
Self-assembly	**-**	1) Dumbbell-like 2) Tadpole-shaped 3) Hexagon 4) Vesicles 5) Snowman-shaped	Crosslinking of polymers through multiple intermolecular forces	1) Simple and effective 2) Easy control of particle shape 3) High degree of order	1) Limited to mass production 2) Condition sensitivity	1) Enzyme-driven 2) Self-driven 3) Composite-driven	[7,32,53,60,61]
Masking	**Sputtering**	1) Star-shaped 2) Spherical 3) Hexagon 4) Tubular	One side of the particles is exposed and modified, the other side remains unchanged	1) Suitable for the preparation of large-volume NPs 2) High-precision control is possible	1) Lower production 2) Reaction conditions are harsh and the process is complex	1) Light-driven 2) Ultrasound-driven 3) Composite-driven	[7,32,53,[62], [63], [64]]
	**Pickering emulsion**	1) Spherical 2) Star-shaped 3) Snowman-shaped	The adsorption and stabilizing effect of solid particles at the oil-water interface	1) Available in different sizes and shapes 2) Wide selection of materials	1) Mass production is not possible 2) Harsh conditions	Enzyme-driven	[7,32,65,66]

### 2.1 Phase separation strategy

**Fig. 1:**
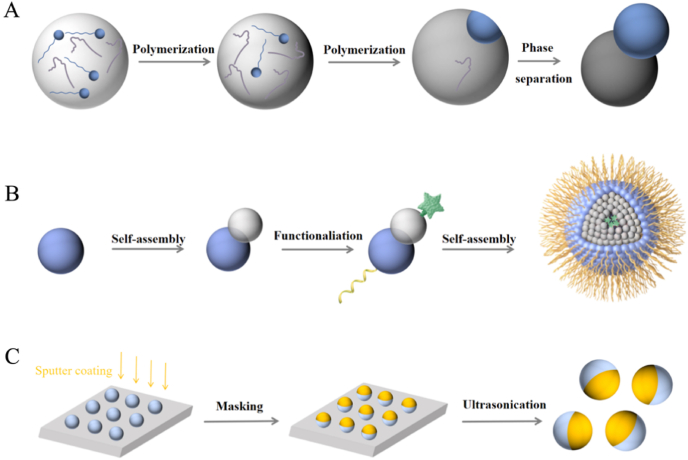
The three major synthesis strategies for JNMs. (A) The phase separation strategy generally consists of three steps: polymerization of substances in the system, polymerization again, phase separation occurs. (B) The self-assembly strategy generally consists of three steps: combining basic units with each other through self-assembly, functionalization modification to give the material a specific function, and self-assembly again to form a complex system with a specific structure and function. (C) The masking strategy generally consists of three steps: deposition of a coating on the surface of a sphere on a substrate by sputter coating, a masking operation to protect a part of the sphere, utilization of ultrasonication to obtain a sphere material with a specific coating distribution.

Phase separation is a strategy of synthesis based on differences in the compatibility of substances. The strategy centers on the mixing of two poorly compatible but miscible substances in a highly volatile solvent. By adjusting the temperature, the solvent is dissolved and evaporated, which in turn induces phase separation and the formation of JNMs with different regionalities (Fig. 1A) [33]. Phase separation strategy mainly includes the microfluidic strategy and the seed-mediated polymerization strategy. Among them, the microfluidic strategy is a new technology in phase separation strategy. Recent studies indicate that microfluidic strategies can produce JNPs with uniform particle size and controllable morphology and composition. Furthermore, by adjusting the flow rates of the two phases, the phase ratio of JNPs can be regulated [7]. Currently, microfluidic strategies have enabled the synthesis of anisotropic magnetic Janus NPs as well as gold NPs [[34], [35], [36]]. Han et al. reported on 2D colloid array technology using Janus microparticles (JMPs) with controlled anisotropic Particle geometry [37]. They prepared JMPs using microfluidic methods and achieved efficient control of their monodispersity, geometric anisotropy, and morphological stability by precisely adjusting fluid parameters. But it relies on fluid viscosity matching (0.087–0.137 Pa s), and excessive viscosity differences may disrupt monodispersity, which may limit its application in extreme structures or diverse fluid systems. In addition, seed-mediated polymerization is also one of the commonly used strategies for phase separation strategy [7]. This strategy is able to precisely construct JNMs with specific structures by triggering the polymerization reaction on the surface of seed particles. Taking the study of Tian et al. as an example, they successfully prepared Janus dimers using seed emulsion polymerization and realized the structural design by regulating the reaction temperature and monomer ratios [38]. The average particle size of PGMA/PSMA Janus dimer is 1.46 μm, and the size of the two regions is about 1.4 μm and 1.1 μm, reflecting its size uniformity. Kim et al. also tracked the morphological changes of Janus NPs prepared *via* seed-mediated polymerization using optical microscopy and found that the particle geometry could be readily altered by adjusting the monomer ratio in the mixture [39].

A further review of the phase separation strategy reveals that it is capable of realizing the construction of complex JNMs by virtue of its unique reaction mechanism, which is an irreplaceable advantage in the customized preparation of micro- and nanostructures. The seed mediated aggregation strategy has the best comprehensive performance in terms of mass production efficiency, cost control, and functionality, and is currently the preferred technological path for JNM's actual large-scale production. Microfluidic strategies are suitable for production with high added value and high precision requirements, and currently do not have the economic feasibility for large-scale promotion [37,38]. Moreover, JNMs produced *via* microfluidic strategies are predominantly micrometer or millimeter in scale. Due to their larger diameters, direct clinical application poses significant challenges [32]. No matter which method is adopted, in the practical application process, it is highly dependent on professional equipment. Both the purchase of equipment in the early stage and the maintenance in the later stage require a relatively high capital investment. Meanwhile, in realizing high-precision particle size control, it is still necessary to break through the limitations of the existing technical parameters to meet the diversified scientific research and industrial needs.

### 2.2 Self-assembly strategy

The self-assembly strategy is a technique that utilizes basic structural elements to spontaneously form ordered structures in solution or at interfaces [40]. In most cases, the first step involves synthesizing a relatively small NP core. Subsequently, by modifying and adjusting the reaction conditions, another material is deposited onto the NP core through polymerization [41]. This strategy makes full use of the physical and chemical properties of the structural units to prepare JNMs of different sizes (Fig. 1B). J. Song et al. used Fe_3_O_4_-Au JNMs as the basis, which formed amphiphilic particles after grafting poly (lipid hydroperoxide) and poly (ethylene glycol), respectively. In solution, the amphiphilic particles first form small micellar clusters, which fuse to form worm-like micelles and eventually assemble into bilayer vesicles [42].

The self-assembly strategy has a significant advantage in the preparation of JNMs with a small particle size. This is due to the fact that the structural units of small-sized particles are easier to achieve an ordered arrangement. However, the construction of JNMs with larger structural units is still at an early stage and is technically difficult [7]. The self-assembly strategy has been widely used in industry, and its main advantages are easy operation and low cost. However, the strategy still needs to be improved in terms of precise control of particle size and shape.

### 2.3 Masking strategy

Masking is a facile strategy of synthesizing JNMs that offers significant flexibility in synthesizing materials [43]. The basic principle is to mask one side of a homogeneous particle with a masking agent, modify the other side physically or chemically, and then remove the masked coating to obtain anisotropic JNMs (Fig. 1C) [44]. The masking strategy mainly include sputtering strategy and Pickering emulsion, *etc.* Among them, the sputtering strategy forms Janus structures by changing the exposed surfaces of uniform particles while the unexposed surfaces remain intact [45]. For example, to form the Janus structure, polymer particles are deposited as a monolayer on the surface of a silicon wafer, and then gold is selectively sprayed on a portion of the particles by the sputter-coating strategy [46]. By controlling the deposition time, it is easy to control the shape and thickness of the metal film, but low yield is unavoidable [47]. Sputtering can be used to prepare most JNMs, provided that uniform particles do not stack on the substrate. In addition to sputtering, other strategies to effectively synthesize JNMs have been invented, such as the Pickering emulsion. The NPs in the pickling emulsion are located at the interface division of two liquids, and this liquid-liquid interface state makes the NPs have two different surfaces in order to be easily modified to prepare two-sided particles [48]. Qiang et al. stabilized oil-in-water Pickering emulsion using modified silica particles. Through microemulsion polymerization, polystyrene selectively nucleated and grew to form mushroom-like polymer JNMs. Overall, the acid-washed emulsion strategy can provide a dual, unique chemical environment for solid NPs [49].

Although masking strategies can precisely adjust the surface properties of particles, they also present certain drawbacks during the clinical translation and large-scale production process. The masking method requires the protection and deprotection of active functional groups in multifunctional compounds at each step, resulting in a complex process that leads to low yields and an enormous workload [50].

The strategies for JNMs synthesis show their own advantages and disadvantages, and researchers need to choose the appropriate one according to the actual needs and application scenarios. Although these traditional preparation methods can realize the preparation of JNMs at present, there are still some problems in large-scale production. For example, the microfluidic method may experience uneven mixing of substances in large-scale production, resulting in a loss of uniformity in the product. In addition to this, the need for the molecules and units used for self-assembly to have specific chemical groups or symmetries greatly limits the generalizability of the approach [6].

In order to solve the deficiencies in the preparation method, improvements can be made in terms of equipment, material selection, and preparation method [51,52]. For example, the introduction of emerging technologies, such as 3D printing, has realized the double improvement of product preparation efficiency and product quality by taking advantage of its precise, controllable, and flexible molding advantages. It should be noted that nanomaterials and printing “ink” are prone to aggregation and reaction due to high surface energy, and may also conflict with processes such as high temperature and light exposure, leading to functional failure. The accuracy of the equipment is difficult to match at the nanoscale, and batch consistency and cost control are challenging when scaling up. With the gradual advancement and practical validation of these improvement strategies, it is expected to further unleash the performance potential of JNMs and promote their innovative applications in the fields of biomedicine and environmental governance to a new level.

## 3 Externally field-driven JNMs

### 3.1 Light-driven JNMs

Light-driven JNMs utilize the properties of their structural materials to generate photothermal, photoelectric, or photochemical reactions that drive their motions. Most of the JNMs have a metallic composition and can utilize NIR to achieve motion through the photothermal effect [46]. Light-driven nanomotors can realize efficient motion in living organisms, advancing efficiently in the form of “auto-thermophoresis”, with the speed of motion increasing with the increase of laser power. At the same time, the light-driven nanomotor can actively approach the target, which enhances the chance of contact with the target [67]. In addition, light-driven nanomotors can be combined with various therapeutic modalities, such as immunotherapy, to achieve multifunctional therapeutic goals (Table 2) [68,69]. Currently, such light-driven JNMs with multifunctionality have been widely used in various disease diagnostic and therapeutic fields, and we will present three aspects of motion penetration, drug delivery, and photothermal ablation.

**Table 2:** JNMs driven by light.

Composition	Size	Photosensitive material	Locomotion mechanism	Light wavelength	Specific usage	Advantages	Remarks	Morphology	Schematic diagram	Ref.
MJAMS/PTX/aV	450 nm	Pt	The photothermal effect produces a temperature gradient	808 nm	AS treatment	1) Photothermal ablation of macrophages 2) Anti-proliferation effect	Combination of photothermal and drug therapy	Irregular spherical		[30]
CHI Hep Au Erythrocyte membrane	–	Au	The photothermal effect produces a temperature gradient	760 nm	thrombus ablation	1) Antibiofouling 2) Efficient thrombolysis	Erythrocyte membrane coating for biomimetic properties	Spherical		[46]
mSiO_2_ Roussin's black salt	36.13 ± 1.08 nm	Roussin's black salt	Roussin's black salt is irradiated with light to release NO	980 nm	AS treatment	1) Enhanced bioavailability 2) Can relieve AS	1) Precise morphology control 2) Customizable asymmetric ratio	Irregular spherical		[77]
J-CeM@Au	>190 nm	Au	The photothermal effect produces a temperature gradient	808 nm	Periodontitis treatment	Highly efficient ROS scavenging	1) Multifunctional integration 2) Enhanced therapeutic efficacy	Spherical		[68]
AMM-Col	234 nm	Au	The photothermal effect produces a temperature gradient	–	Deep tumor penetration and enhanced tumor immunotherapy	1) High photothermal stability 2) Excellent motility	Synergistic deep tumor penetration	Irregular spherical		[69]
SiO_2_ Au D-RK10-Cys	106 nm	Au	The photothermal effect produces a temperature gradient	800 nm 808 nm	Prevention and Treatment of Alzheimer's Disease	Can improve blood-brain barrier penetration	Avoids harsh conditions of other therapies	Spherical		[67]
GNR@PAA - Mn@COS	145 ± 20 nm	Au	The photothermal effect produces a temperature gradient	808 nm	Cancer Therapy	1) pH and NIR-responsive drug release 2) Enhanced targeting to tumor cells	Active targeting ability enhanced by COS	Irregular spherical		[78]
Dox/HCPT loaded UFO-like CD-PdNS/ZIF-8 JNP	>45 nm	Pd	The photothermal effect produces a temperature gradient	1064 nm	Synergistic cancer therapy	1) pH and NIR-II stimuli-responsive drug release 2) Excellent photothermal performance and deep tissue penetration ability	First-time fabrication by tailoring the surfaces of 2D PdNS	UFO-like		[79]

#### 3.1.1 Motion penetration

Infrared light has a certain energy, and light in the NIR region of 650–950 nm can penetrate tissues, which implies that NIR-propelled JNMs theoretically have good penetration capabilities. Various reports have now shown that NIR light-propelled JNMs can perform complex *in vivo* tasks such as thrombosis, deep tumor penetration, and cell membrane penetration [46,70,71].

In addition to being widely used in cardiovascular disease treatment and cancer therapy, NIR-driven JNMs have also emerged in some neurological and immune system diseases in recent years. In the treatment of Alzheimer's disease, Liu's team developed inhibitor-coupled NIR-driven JNM (JNM-1) [67]. Blood-brain barrier is a major challenge for drug delivery in neurodegenerative diseases, and JNM-1, driven by NIR, has a higher blood-brain barrier penetration ability due to its photothermal effect and active motility properties. They carried out the study by constructing an *in vitro* blood-brain barrier model, in which brain endothelial cells (HAECs) were inoculated in cell plates to which FITC-labeled JNM-1 was added, and some of the samples were irradiated with 808 nm laser light for 10 min, and the unirradiated samples were used as controls. The results showed that the green fluorescence signal was significantly enhanced in the JNM-1 group treated with NIR irradiation compared to the control group. This indicates that NIR irradiation effectively improved the penetration ability of JNM-1, making it easier to cross the blood-brain barrier.

**Fig. 2:**
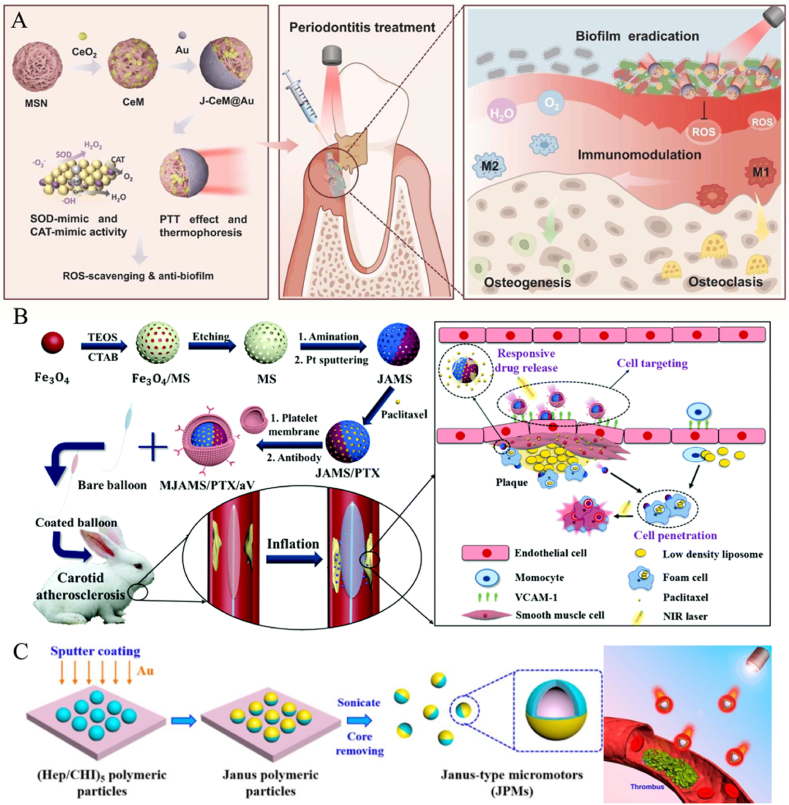
(A) Preparation process of J-CeM@Au and mechanism of treatment of periodontitis. (B) Synthesizing process of MJAMS/PTX/aV and the mechanism of anti-AS. (C) Synthesis process of EM-JPMs and the mechanism of photothermal thrombolysis. Reproduced with permission from Refs. [30,46,68].

In the field of periodontitis treatment, biofilms formed by deep-seated pathogens are difficult to remove by conventional means due to their complex structure [72]. Therefore, Bai et al. used CeO_2_-loaded mesoporous SiO_2_ as a substrate and coated gold NPs on its surface by sputtering to produce the JNM (J-CeM@Au) for periodontitis treatment, to utilize its penetration and antibacterial properties (Fig. 2A) [68]. Using mesoporous silica loaded with CeO_2_ as the substrate, AuNPs were deposited only on half of the CeM particle surface, forming an asymmetric Janus structure. In order to verify its penetration performance, a two-species biofilm composed of *Porphyromonas gingivalis (P. g)* and *Clostridium nucleatum (F. n)*, which causes periodontitis, was used as a model, and the biofilm was irradiated with NIR for 5 min after the addition of a suspension of fluorescently labeled NPs. The results of the Confocal Laser Scanning Microscope observations showed that, in the absence of NIR irradiation, only weak red color fluorescence was observed in the biofilm of the control group; and in the absence of NIR irradiation, only weak red color fluorescence was observed in the control group. Fluorescence; while after NIR laser irradiation, strong fluorescence appeared, and the J-CeM@Au nanomotors could penetrate almost the whole biofilm layer.

Single-atom catalysts have garnered attention in cancer therapy due to their high catalytic activity, but most lack self-propulsion capabilities, making it difficult to actively approach cancer cells or penetrate tumor interiors. To address these challenges, Xing et al. combined single-atom nanocatalytic drugs with nano-engines, designing nitrogen-doped jellyfish-like mesoporous carbon nano-engines (Cu-JMCNs) to enhance tumor treatment [73]. Cu-JMCNs exhibit a biomimetic jellyfish-like asymmetric structure: the “bell-shaped” main body features mesopores on its surface, while the “arm-like” protrusions form non-porous branched structures. Copper is uniformly anchored to the carbon framework through coordination with pyridine nitrogen. Its propulsion relies on carbon components absorbing NIR light, generating a non-uniform thermal distribution within the asymmetric structure. This creates a temperature gradient that drives the nano-engine's directional motion. Experiments demonstrate that without NIR light, Cu-JMCNs undergo passive diffusion only to the surface layer. After 30 min of 0.5 W/cm^2^ NIR irradiation, penetration reaches 40–80 μm depth, increasing tumor inhibition by 38.6 %. *In vivo* experiments show that Cu-JMCNs propelled by NIR light achieve an 85 % tumor inhibition rate.

#### 3.1.2 Drug delivery

The NIR driver gives JNM good penetration ability, enabling it to break through the biological barrier and reach the lesion site. This feature creates favorable conditions for the precise release of drugs at the target site, while the effectiveness of drug delivery depends on the motion penetration ability of JNM. Therefore, there is a complementary relationship between the motor penetration and drug delivery of JNM, and the synergistic effect of the two together promotes the development of nanomotor applications in the field of disease treatment.

Atherosclerosis (AS) is a chronic inflammatory disease in which fatty deposits accumulate in the walls of blood vessels, leading to reduced blood flow [74]. To treat AS, Huang et al. prepared an aminated silica (AMS) with an ordered mesoporous structure for drug-carrying and slow-release, and then made Janus AMS (JAMS), which can penetrate deep into the plaque, by sputtering metallic platinum to form an asymmetric structure on one side of AMS. On this basis, anti-proliferative drug *paclitaxel* (PTX) and anti-vascular cell adhesion molecule-1 (anti-VCAM-1) antibodies were modified and wrapped around the platelet membrane to prepare MJAMS/PTX/aV (Fig. 2B) [30]. They showed that the motion curve of JAMS caused by NIR conformed to the quadratic equation, so it was light-driven autonomous motion rather than Brownian motion. At the same time, they examined the Pt content in the blood clot model by testing the Pt content. The results showed that the residual rate of JAMS within the model was nearly 13 times higher under NIR conditions than under non-NIR light, implying that the movement of JAMS by NIR light irradiation may contribute to their retention and penetration at the plaque site, which lays the foundation for drug release from the lesion site. In addition to the results achieved in the treatment of atherosclerotic disease, JNM's explorations in cancer drug delivery are equally exciting. For example, Ma et al. constructed a NIR-driven drug-carrying JNMs, in which collagenase-coated gold nanorods were connected to chitosan-functionalized mesoporous silica *via* Mg^2+^, which could effectively penetrate tumors for drug delivery [69].

Gene therapy represents a crucial approach for treating critical limb ischemia (CLI), yet traditional delivery systems face limitations in passive distribution and the ischemic microenvironment barrier, hindering efficient therapeutic gene delivery. Addressing this challenge, Gui et al. designed a NIR light-driven Janus poly(dopamine)@mesoporous silica gene delivery nanomotor (PDA@MS-NH_2_@HIF-1α), offering a novel therapeutic strategy [75]. This nanomotor exhibits an asymmetric yolk@shell Janus structure, with a PDA core and a mesoporous silica shell. Within this Janus structure, the asymmetric distribution of PDA yolk provides the foundation for photothermal actuation. Driven by low-energy NIR laser irradiation, the motor's superior photothermal properties generate a localized asymmetric temperature gradient around it, propelling directed motion. *In vitro* hypoxic conditions, this nanomotor significantly enhanced HIF-1α/VEGF protein expression in human aortic HAECs. In an *in vivo* mouse hindlimb ischemia model, intramuscular injection combined with NIR irradiation significantly restored blood flow in the ischemic limb.

Therapeutic angiogenesis represents a critical intervention for CLI. However, the high toxicity of free Cu^2+^ and the low delivery efficiency of traditional carriers have hindered its application. Addressing this, Gui et al. designed a NIR light-driven copper-loaded PDA nanomotor (JMPN@Cu^2+^), offering a novel therapeutic pathway for CLI [76]. Similar to Xing et al.'s material, this nanomotor exhibits a jellyfish-like Janus structure. However, its core consists of a PDAscaffold, with Cu^2+^ loaded *via* chelation with PDA phenolic hydroxyl groups. Under 808 nm laser irradiation (1.0 W/cm^2^), PDA generates a local temperature gradient, driving the motor's directional motion with performance significantly superior to Brownian motion. *In vitro* under hypoxic conditions, 4 μg/mL JMPN@Cu^2+^ combined with NIR irradiation achieved a migration rate of 76 % in human aortic HAECs. *In vivo*, in a mouse hindlimb ischemia model, intramuscular injection for 28 d resulted in an ischemic limb perfusion ratio of 0.912 ± 0.124, with a significant increase in CD31-positive vascular density.

#### 3.1.3 Photothermal ablation

In addition to motion and drug delivery, the NIR-driven JNM can also function as a photothermal ablator for the photothermal ablation of blood clots. Therefore, Shao et al. prepared Janus-type polymeric micromotors with an erythrocyte membrane (EM-JPMs) by self-assembly of alternating coatings of polysaccharides and heparin adsorbed on the outer surface of silica, on which the particles were partially coated with gold with an outer erythrocyte membrane (Fig. 2C) [46]. Due to the asymmetric distribution of the gold, the localized thermal gradient generated by NIR light irradiation could produce motion by the autothermic swimming effect. Particle motion videos show a complete cessation of particle motion when the NIR laser is turned off and an immediate restart of motion when it is turned on. The researchers analyzed the thrombolytic effect of EM-JPMs using a Confocal Laser Scanning Microscope, and time-lapse Confocal Laser Scanning Microscope images showed that EM-JPMs with red fluorescence irradiated with the NIR laser could disrupt the green fluorescence-stained fibrin network representing the thrombus, resulting in a gradual release of fluorescence from the thrombus and spreading it to the neighboring regions. The experiment also set up several control groups, such as EM-JPMs prepared by replacing Hep with sodium alginate, a laser irradiation group without EM-JPMs, and a (Chitosan/Heparin)_5_ capsule group without gold coating, which were unable to induce thrombus ablation, highlighting the critical roles of Hep and gold coating in the photothermal ablation of thrombus by EM-JPMs.

Although light-driven nanomotors provide useful ideas for the treatment of a variety of diseases, they also have certain problems. First, JNMs rely on specific photosensitive materials to realize the light-driven function, and most of these photosensitive materials are metals. The power is obtained by irradiating light so that it produces a photothermal effect and forms a temperature gradient. This limits the range of material choices to some extent. Second, the absorption and scattering of light by biological tissues will weaken the intensity of light, resulting in the attenuation of its energy in the penetration process, so when treating some tumors located in the deep tissues of the human body, it is difficult for the light to reach the target area sufficiently to play a role. Third, the photothermal effect may have adverse effects on the surrounding normal tissues and may also trigger local inflammatory reactions, resulting in side effects [67]. In conclusion, we need to fully consider the advantages and disadvantages of light-driven nanomotors to better promote their clinical applications.

### 3.2 Magnetically-driven JNMs

In addition to the above driving methods, prompting particle motion by means of the interaction between the magnetic field and JNMs is becoming a new focus of research in the field of nanomotor driving, and this driving strategy provides a brand new perspective for motion control at the nanoscale. Magnetic field actuation can be controlled with high precision, and by precisely controlling the change of the magnetic field, precise control and positioning of the controlled object can be achieved. In addition to this, magnetic actuation has good advantages in increasing penetration. Due to the magnetic component in JNM, it can penetrate the tissues under the effect of an alternating magnetic field and realize magnetothermal therapy [58,80]. Magnetic actuation not only enhances penetration but also realizes the aggregation of JNM at the lesion site, better enabling its application for drug-targeted delivery. When JNM possesses magnetic properties, it can deliver drug targeting to the lesion site under the guidance of the magnetic field to improve the drug therapeutic effect [81]. This new type of NP achieves directional actuation of JNMs under the action of a magnetic field by asymmetrically combining magnetic metals or particles onto the surface of the NPs (Table 3) [82].

**Table 3:** JNMs driven by magnetism.

Composition	Size	Magnetic material	Magnetic field intensity	Specific usage	Advantages	Remarks	Morphology	Schematic diagram	Ref.
PEMP	20 μm	Ni	1.8 T 10 mT	Treatment of neurological diseases	1) Low-intensity ultrasound requirement 2) High-frequency neuromodulation	Effective neural stimulation under low-intensity focused ultrasound	Spherical		[93]
Regorafenib PCL Doxorubicin PLGA	120 μm	Iron oxide	7 T	Combination chemotherapy for hepatocellular carcinoma	1) Encapsulation of multiple drugs 2) Synergistic anticancer effect	Phase-separated dual-compartment for drug loading	Snowman-shaped		[20]
SiO_2_ Fe_3_O_4_ APTES Succinic anhydride NHS EDC Antibodies	–	Fe_3_O_4_	–	Noninvasive detection of bladder cancer-derived urine exosomes	1) High sensitivity 2) Good specificity 3) Multiplex detection	1) Structural color encoding 2) Noninvasive analysis	Spherical		[87]
M-MSNs	–	Iron-based	325 Oe 262 kHz	Liver cancer	1) Superior superparamagnetic properties 2) High curcumin loading ability	The combination of chemotherapy and magnetic hyperthermia therapy (MHT)	A spherical magnetic part and a rod- like silica part		[80]
Fe_3_O_4_@PFODBT-COOH	42.3 nm	Fe_3_O_4_	–	*In vivo* cell tracking	1) High MPI signal intensity 2) Multimodality imaging	High sensitivity in MPI	Spherical		[94]

**Fig. 3:**
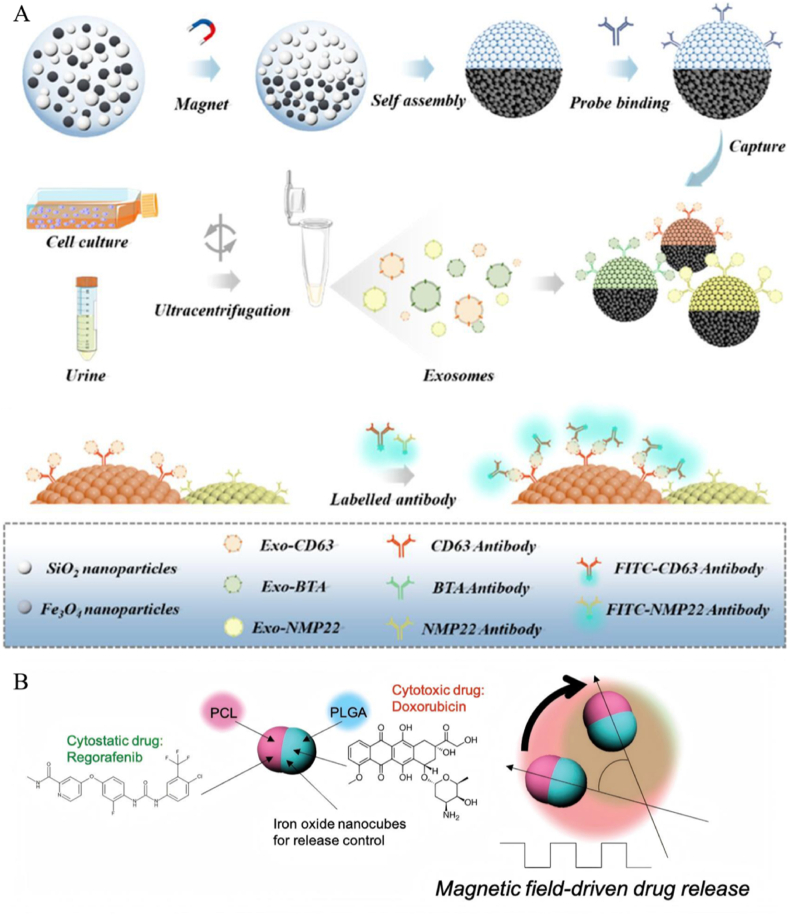
(A) Preparation of Janus magnetic beads and a multiplex exosome analysis platform based on the barcoding of Janus magnetic beads. (B) Schematic diagram of the experimental setup for magnetic field-triggered drug release from Janus microcarriers and anticancer effects on human hepatocellular carcinoma cell lines. Reproduced with permission from Ref. [20]. Reprinted with permission from Ref. [87]. Copyright 2022 American Chemical Society.

The efficiency of JNMs to reach the diseased area can be improved by the magnetic force generated by the magnetic field on JNMs. In recent years, bladder cancer has become one of the most common malignant tumors in the urinary system, posing a great threat to people's life and health [83]. The examination of tumor markers to determine the presence of a tumor is now a common method of tumor diagnosis. Among the various tumor markers, exosomes are one of the most promising indicators for the diagnosis of bladder cancer [84,85]. Current methods of exosome detection usually require the collection of tissue specimens or blood samples, but this is invasive causing patient discomfort, and conventional analytical platforms can only identify a single type of exosome [86]. Therefore, there is an urgent need for a new non-invasive and multi-analysis platform. Wei et al. prepared a multiplexed assay platform based on Janus magnetic photonic crystal microsphere barcodes (Fig. 3A) [87]. These magnetic microspheres have a Janus structure, with one half being magnetically responsive and the other half displaying the characteristic structural color of the coding element. The characteristic structural colors of the microspheres enable barcode analysis of foreign bodies, while the magnetic responsiveness of the microspheres facilitates their controlled rotation in the sample solution, thus improving the enrichment efficiency and sensitivity of the assay. To achieve multiple analyses of this magnetic microsphere, the researchers chose Janus magnetic microspheres with 565, 595, and 610 nm reflection peaks to be attached to several different antibodies, respectively. These probe-conjugated microspheres were incubated with bladder cancer exosome samples, and then Fluorescein isothiocyanate-labeled multiple different antibodies were added to the detection solution for reaction. At the end of the reaction, the Janus magnetic microspheres were characterized by fluorescence imaging, which was completed by analyzing the characteristic spectra using fluorescence microscopy to decode the Janus barcode.

Besides bladder cancer, liver cancer is one of the most common and highly lethal cancers in the world. Chemotherapy is commonly used in the treatment of hepatocellular carcinoma, but existing chemotherapy is unsatisfactory due to severe intrinsic resistance and systemic toxicity. Therefore, Janus microcarriers have been developed for the treatment of hepatocellular carcinoma. Cho's team used polycaprolactone (PCL) compartments and magnetic NP-loaded poly (propylene glycolide-co-glycolic acid) (PLGA) compartments to form the Janus microcarrier, with the two compartments containing hydrophobic regorafenib and hydrophilic doxorubicin, respectively (Fig. 3B) [20]. We achieved the combined release of dual chemotherapeutic agents by exploiting magnetic anisotropy and controlling the movement of the Janus microcarriers with a magnetic field. It has been shown that the frequency of the magnetic field can directly affect the release of both chemotherapeutic agents. When the frequency of the alternating magnetic field changes, the rotation angle and angular velocity of the microcarrier change. In the range of 0–100 mHz, the angular velocity increases with the increase of frequency; above 100 mHz, the rotation angle decreases, and the motion tends to be close to vibration. 100 mHz has a larger angle of rotation and the highest angular velocity of the microcarrier, which is the most active rotation of the microcarrier at this time. The drug release was related to the rotational motion of the microcarriers. JNMs showed the highest release rates for both regorafenib and doxorubicin at a frequency of 100 mHz, releasing up to 32 % and 72 % from the microcarriers, respectively. And the data showed that released anti-angiogenic regorafenib and cytotoxic doxorubicin promoted apoptosis and reduced micro-vessel development in TdT-mediated dUTP Nick-End Labeling-positive cells in hepatocellular tumor regions, including the border region. This suggests that magnetic fields can not only drive JNMs but also enhance the therapeutic effect of the Janus system by controlling the amount of drug released. Recent clinical trials have also involved magnetic tumor-targeting nanomaterials [[88], [89], [90], [91]].

In practice, magnetic field generation, control, and precision focusing techniques limit the use of magnetically driven JNMs in some environments. By constructing a programmable magnetic field array and utilizing multiple small electromagnetic coils to form an array, the current magnitude and direction of each coil can be precisely controlled, thereby achieving flexible regulation of magnetic field distribution. This method can improve the precise focusing ability of the magnetic field, generate highly concentrated magnetic fields for specific areas, and reduce the impact on surrounding tissues [92]. This method can have a certain impact on the improvement of traditional methods. In terms of health risks, while some magnetic materials are considered biocompatible to some extent, the potential risks at long-term or high-dose exposures are unclear.

### 3.3 Ultrasound-driven JNMs

The ultrasound-driven JNM is a nanoscale power device based on an asymmetric structure and ultrasonic energy input. Due to its excellent biocompatibility and controllability, ultrasound-driven JNMs are widely used in liquid environments, such as thrombus sites and cancer cells [22,23]. Based on its non-invasive, highly controllable, and chemical-fuel-free characteristics, it enables it to reduce the damage to normal tissue cells, and is able to enhance therapeutic efficacy by performing thrombus removal and direct killing of tumor cells with minimal damage.

**Fig. 4:**
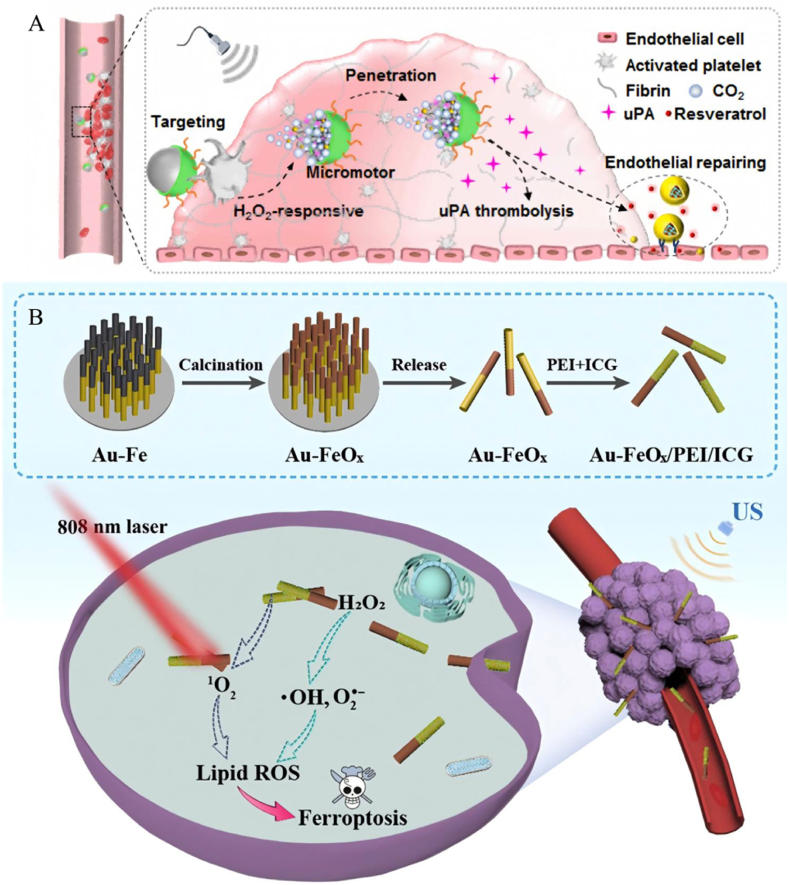
(A) rJPox@u-HARes MPs thrombolysis mechanism diagram. (B) Schematic diagram of the preparation of AFPI NMs and their induced ferroptosis in cancer cells. Reproduced with permission from Refs. [22,23].

Regarding the treatment of thrombus, an ultrasound-driven JNM has been designed for efficient thrombolysis and inhibition of thrombus recurrence. Cao's research team prepared a new type of microelectrode for the treatment of thrombus by encapsulating urokinase-type plasminogen activator and resveratrol-loaded hyaluronic acid NPs in hydrogen peroxide-sensitized poly (1,4-cyclohexanedimethanol-oxalate) particles and prepared JNMs with targeting and stimulus responsiveness by PDA capping and RGD grafting (Fig. 4A) [22]. The design principle of this micromotor is to utilize the high concentration of H_2_O_2_ at the thrombus site to trigger the oxidative degradation of POX to produce CO_2_ [22]. Under the action of ultrasound, the cavitation effect of CO_2_ bubbles can drive the micromotor to move and enhance its penetration ability in the thrombus, thus accelerating the release of urokinase-type plasminogen activatorurokinase-type plasminogen activator and resveratrol-loaded hyaluronic acid NPs and realizing the dual functions of targeted thrombolysis and endothelial repair.

In the field of tumor therapy, ultrasound-driven JNMs have demonstrated unique advantages in promoting iron death (ferroptosis) in cancer cells. The nanomotor designed by Chen et al. consists of gold-iron oxide rod-shaped bimetallic nanomotors (Au-FeO_x_, AF NMs) and a photoactive indocyanine green dye on the surface, composed of (Fig. 4B) [22]. This motor is based on mesoporous titanium dioxide (TiO_2_) NPs, which are asymmetrically coated with a thin silver layer and loaded with L-arginine (LA) to form a Janus structure. Ultrasound can rapidly internalize this nanomotor into cancer cells with a four-fold increase in cell internalization efficiency. This is due to the fact that under specific ultrasonic conditions, the rapid movement of AF NMs provides sufficient momentum for their contact with cancer cells, enabling the nanomotor to effectively break through the microenvironmental obstacles around the cell and make close contact with the cancer cells, thus creating conditions for entry into the cell. This actively movable nanomotor is 88 % more efficient in killing cancer cells than stationary nanomotors. Unlike previous passive strategies, this work is an important step forward in accelerating cellular endocytosis and inducing iron death in cancer cells in an active manner, which presents us with an aggressive approach to treating cancer using ultrasound-driven therapy.

In summary, ultrasound-driven has shown some advantages in cancer treatment and thrombosis therapy, but there are also drawbacks. In cancer therapy, when ultrasound-driven JNM is used to internalize cancer cells, the high intensity and prolonged action may damage other healthy cells, and in practice, it is difficult to control it precisely so that it reaches the position and is released at the right time [22]. In the field of thrombosis, ultrasound alone has a limited thrombolytic effect, and high-intensity focused ultrasound can cause vascular damage [95]. Moreover, the ultrasound-driven system is limited by potential off-target effects. Constrained by the precision of ultrasound energy focusing, energy tends to deviate from the target area and act on normal tissues, leading to adverse consequences such as cellular damage and tissue inflammation. Simultaneously, variations in acoustic impedance and density among different tissues within the body can cause unintended deposition of ultrasound energy in non-target regions, further exacerbating the risk of off-target effects.

### 3.4 Electric-drive JNMs

Electric fields have garnered attention for their ability to efficiently power moving objects over long distances. By adjusting the amplitude and frequency of alternating current (AC) signals, these fields can be precisely controlled. Electrically driven JNMs utilize AC electric fields to induce surface asymmetric charge separation within metal-dielectric asymmetric structures, generating dielectrophoretic forces that convert electrical energy into directed mechanical motion. Among these, Janus-structured motors can precisely concentrate in target regions *via* dielectrophoretic forces, enhancing targeting and controllability. Based on these characteristics, electrically driven JNMs offer significant advantages in medical applications. They do not rely on toxic chemical fuels, operating solely on external AC electric fields. Their core materials exhibit high biocompatibility, enabling safe operation within physiological fluids. Furthermore, Janus structures can integrate drug delivery, imaging, and sensing functions while overcoming physiological barriers like dense tumor stroma, making them well-suited for targeted therapy and integrated diagnosis-treatment medical needs.

**Fig. 5:**
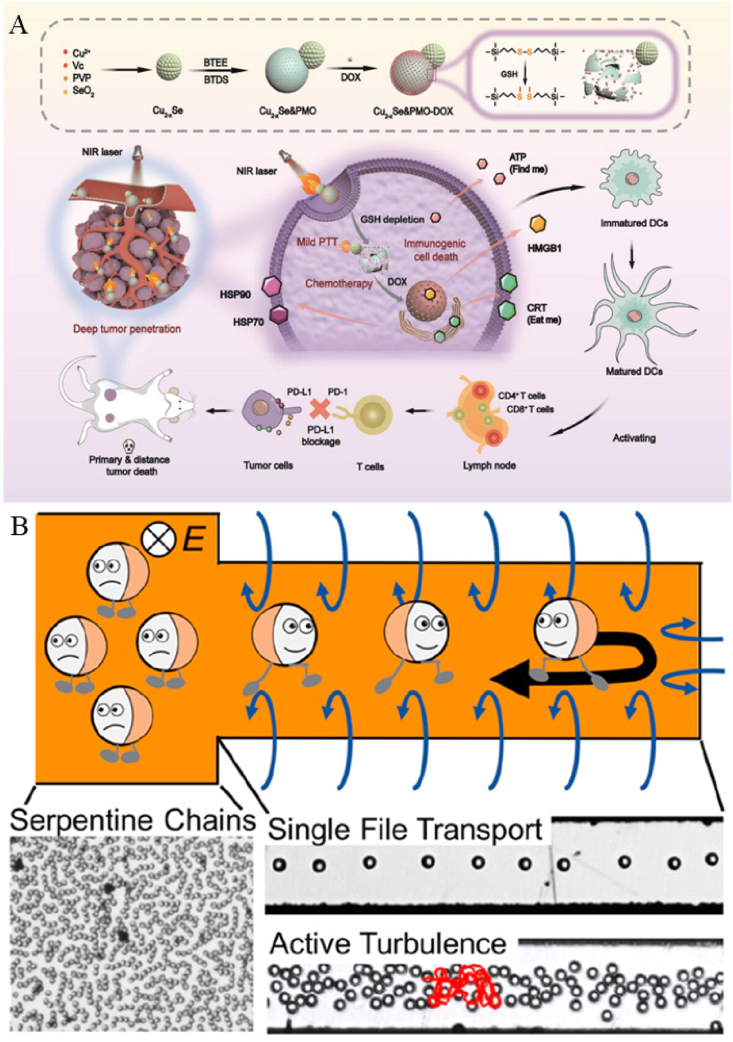
(A) Schematic diagram of the preparation and mechanism of metal-dielectric composite electric-driven JNMs; (B) Schematic diagram of the mechanism of action of SiO_2_-Ti JNMs. Reprinted with permission from Refs. [96,98].

In the treatment of triple-negative breast cancer (TNBC), the dense extracellular matrix and abnormal vascular architecture of tumors limit the penetration depth of therapeutic agents. Conventional nanomedicines often accumulate at the tumor periphery, struggling to reach the hypoxic core regions. Electro-driven Janus nano-motors (JNMs), leveraging their chemical-fuel-free operation and highly controllable motion, offer an innovative solution to overcome tumor barriers. Leveraging these properties, Chen et al. developed metal-dielectric composite electric-driven JNMs. Using mesoporous silica as the substrate, they sequentially sputter-coated a Ti transition layer and an Au conductive layer onto the hemispherical particle surface to construct a Janus asymmetric structure (Fig. 5A) [96]. This structure achieves semi-enclosed anisotropic morphology through surface energy regulation, ensuring differential charge polarization. In simulated tumor interstitial fluid between parallel electrodes, these JNMs are driven by contact charge electrophoresis: applying a constant voltage of 200–1000 V causes rapid charge reversal upon particle-electrode contact, inducing rotation that converts into translational motion. Experiments demonstrate that at 400 V and 200 μm electrode spacing, mSiO_2_-based JNMs exhibit an oscillation frequency of 29 Hz and a velocity of 25 μm/s perpendicular to the electric field. When the electrode spacing is reduced to 50 μm, the velocity peaks at 600 μm/s. The effective diffusion coefficient increases threefold compared to the no-electric-field group, with sustained motion exceeding hundreds of micrometers, confirming its highly efficient mobility.

Meanwhile, self-regulation can be easily achieved by adjusting the amplitude and frequency of the alternating electric field signal. The metal-dielectric composite JNMs studied by Dou et al., similar to those by Chen et al., are constructed with SiO_2_ or fluorescent polystyrene as the dielectric substrate. A Ti transition layer and an Au conductive layer are sequentially sputtered onto the hemispherical surface of the particles to form a Janus-like asymmetric structure [97]. When driven by contact charge electrophoresis in mineral oil between parallel electrodes under constant voltage, these JNMs induce particle rotation and convert it into directed motion. Experiments demonstrate that at 400 V and 200 μm electrode spacing, SiO_2_-based JNMs exhibit an oscillation frequency of 29 Hz and a vertical electric field velocity of 25 μm/s. At 50 μm spacing, the velocity peaks at 600 μm/s with sustained motion exceeding hundreds of micrometers, confirming their high-efficiency motion capability. Furthermore, Zhang et al. developed SiO_2_-Ti JNMs by depositing Ti *via* electron beam evaporation onto hemispherical SiO_2_ microspheres to create an asymmetric structure (Fig. 5B) [98]. Driven by an alternating electric field on interdigitated microelectrodes, these JNMs exhibited directional motion: a horizontal electric field confined them to the electrode center *via* induced electroosmotic flow, while a vertical electric field enabled directional movement through induced charge electrophoresis. Experiments demonstrated that at 10 kHz frequency and 2 V_p-p_ voltage, JNMs' speed increased quadratically with voltage. They could travel in a single file along the electrode centerline and automatically turn at closed electrode ends, confirming their controllability and adaptability.

## 4 Fuel-driven JNMs

### 4.1 Enzyme-driven JNMs

Since enzymatic reactions in the human body have a variety of different substrates, the unique design of JNMs propelled by a single enzymatic reaction holds great promise for solving biomedical problems (Table 4). JNMs encounter a series of biological barriers during delivery, and the energy converted by enzymatic reactions can provide powerful energy for JNMs to autonomously penetrate various biological barriers to overcome these delivery barriers [99]. Enzyme-driven nanomotors have several advantages. Enzyme-driven JNMs are mostly made of natural biomaterials, which are biocompatible and safe for application in living organisms [100,101]. In addition, enzyme-driven JNMs utilize biofuels to provide power and have a wider range of reaction substrates to choose from, *e.g.*, urea feeds JNMs with urease, and glucose feeds them with glucose oxidase [100,102]. In addition to the properties of the enzyme itself, the application in the organism is supposed to be a favorable environment, with constant temperature and mild pH of the organism, which is suitable for enzymatic reactions, which lays the foundation for the enzyme to function.

**Table 4:** JNMs driven by enzyme.

Composition	Size	Types of enzymes	Substrate type	*Vivo* tissue environment	Specific usage	Advantages	Remarks	Morphology	Schematic diagram	Ref.
GPNP-PAs	280 nm	GOx	Glucose	Thrombus site	Treating thrombotic diseases	1) Improved targeting ability to thrombus 2) Enhanced thrombolytic effects	PM-camouflaged design	Irregular spherical		[102]
Hap@MSN Au Urease Hyaluronic acid	631 ± 76 nm	Urease enzyme	Urea	Tumor tissue environment	Antitumor therapy	1) HA-mediated enhanced cellular uptake 2) Sustained release in tumor matrix	Intratumoral depot-based delivery	Spherical		[99]
Platelets Urease PLL Sulfo-NHS–biotin Streptavidin	1.4–2.6 μm	Urease enzyme	Urea	Tissue sites associated with cancer cells and bacteria	Anticancer and antibacterial drug delivery	1) High drug loading efficiency 2) Enhanced binding efficiency	Preserving platelet biofunctionality	Spherical		[100]
Magnetic iron oxide Antibodies Catalase Avidin EDC Sulfo–NHS BSA	1 μm	Catalase	H_2_O_2_	Total blood environment	Detecting sepsis biomarkers in whole blood	1) High surface area for rapid target capture 2) Short assay time	1) Can detect target in whole blood within 13 min 2) High sensitivity with low limit of detection	–		[112]
JHP-urease	2.32 ± 0.03 μm	Urease enzyme	Urea	–	Targeted Drug Delivery	1) Controllable motion 2) Multiple cargo loading	1) Complete motion control 2) Reversible velocity control	Hollow sphere		[113]
Au-mesoporous silica	1148 nm	Catalase	H_2_O_2_	Cancer cell intracellular environment	Drug delivery	1) High loading capacity 2) Versatile fabrication process	Enhanced cargo delivery in the presence of fuel and GSH	Hybrid anisotropic nanostructure		[114]
IO@PMB-SNO	233 nm	–	GSH	*In vivo* organization of infected burn wounds	Infected burn wounds treatment	1) Effective biofilm infiltration 2) Non-antibiotic antibacterial effect	Synergistic antibacterial effect of NO and PMB	Irregular spherical		[110]
DNase/PEG-Au-PAA/mSiO_2_	167.8 ± 15.8 nm	DNase	DNA	Tumor tissue environment	Tumor diagnosis and therapy	1) Can work with ultralow DNA levels 2) Responsive to disease-associated DNA gradients	Self-navigating and self-targeting towards apoptotic tumor cells	Asymmetric peanut shape		[111]
Hap MSN DOX Urease Hyaluronidase	Calibr: 45 nm Length: 240 nm	Urease enzyme HAase	Urea HA	Tumor tissue environment	Cancer chemotherapy	1) Prolong blood circulation 2) Enhance extravasation and tumor accumulation	High antitumor efficacy without system toxicity	Rod-like		[28]
PLA Poly (styrene-co-maleic anhydride) Urease Folate DOX	Calibr: 550 nm Length: 1.1 μm	Urease enzyme	Urea	Tumor tissue environment	Cancer treatment	1) Prolong blood circulation 2) Increase tumor accumulation 3) Promote cell internalization	1) Folate-mediated targeting 2) High antitumor efficacy with low toxicity	Rod-like		[9]
HA-JNPs@Cef	302.4 nm	Fhn NPs (Catalase-like activity)	H_2_O_2_	Lung tissue infected with Streptococcus pneumoniae	Pneumonia therapy caused by streptococcus pneumoniae	1) High drug-loading capacity 2) Can decompose H_2_O_2_	Antibacterial and tissue-protective effects	Snowman-shaped		[101]
Spiky Au/mSiO_2_-DAO/Catalase	220.6 ± 1.75 nm	DAO Catalase	Histamine	Immune cell	Potential for immune disease treatment	Sensitive to histamine concentration	Tandem catalytic reaction of dual enzymes	Spiky Janus structure		[115]
MSNs GOx Catalase EDC NHS	800 nm	GOx Catalase	Glucose	–	Drug delivery in general medical treatments	Using biocompatible fuel (glucose)	Demonstrated the assembly and enhanced diffusion of sub-micron-sized JNMs	–		[116]
MSNs GOx INV NHS EDC	1215 nm	GOx INV	Sucrose	In HeLa cancer cells	Cancer treatment	The use of enzymes as control elements allows for a highly specific response to biological molecules	First-time assembly of a Janus nanodevice controlled by a bi- enzymatic cascade mechanism for on-command delivery	Spherical		[117]

#### 4.1.1 JNMs driven by a single enzyme

**Fig. 6:**
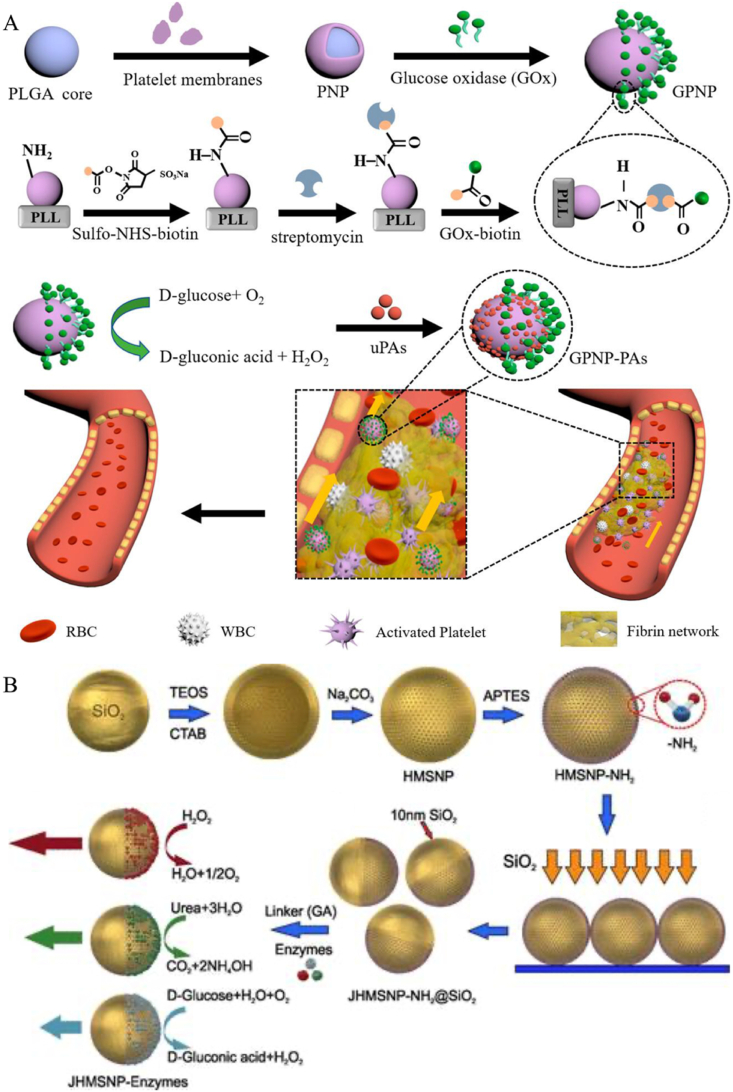
(A) Schematic diagram of the preparation process of GPNP-PAs and its application in thrombolytic therapy. (B) Preparation and mechanism of movement of enzymatically promoted hollow mesoporous silica nanomotors. Reproduced with permission from Ref. [26]. Reprinted with permission from Ref. [102]. Copyright 2023 American Chemical Society.

Glucose oxidase (GOx)-based JNMs are the most common enzyme-driven JNMs. Venous thromboembolism is a complex disease caused by a number of factors, and this disease is one of the leading causes of death in the elderly [103,104]. Urokinase-type fibrinogen activators are widely used and familiar thrombolytic drugs that are better able to promote thrombolysis. To ensure drug delivery to the embolization site, Fang et al. designed multifunctional glucose oxidase (Gox)-based Janus NPs that deliver thrombolytic drugs to thrombosis sites *via* Janus particle motion. In this study, they asymmetrically conjugated Gox to platelet membrane (PM)-coated PLGA NPs, thereby developing a novel Janus nanomotor (GPNP-PA) (Fig. 6A) [102]. In this study, they asymmetrically attached GOx to polymeric NPs coated with platelet membranes (PMs) to develop a novel JNM (GPNP-PA). They produced GOx-biotin by mixing Sulfo-NHS-biotin with GOx solution. This step chemically modified GOx to take on a biotin moiety, creating the conditions for subsequent specific binding to other molecules. To further achieve asymmetric loading, PNPs were added to 12-well plates modified with polylysine, followed by the sequential addition of Sulfo-NHS-biotin, Streptavidin, and GOx-biotin. They utilized polylysine to make the PNPs partially surface-attached, and with the help of the biotin-streptavidin-biotin assay, they allowed the GOx modification to the exposed platelet membranes. The nanomotor undergoes three phases of targeting, penetration, and thrombolysis sequentially after entering the bloodstream. Firstly, the nanomotor modified by PM was able to achieve targeted aggregation of NPs. Secondly, GPNP-PA in the presence of glucose was asymmetrically bio-catalytically decomposed to hydrogen peroxide and gluconic acid by GOx due to the asymmetric distribution of GOx on the platelet surface. This induces a concentration gradient and directional convection of reaction products around the nanomotor, which propels the nanomotor forward on its own. Finally, after the nanomotor aggregates at the thrombus site, the thrombolytic drug Urokinase-type fibrinogen activators exerts its thrombolytic effect to dissolve the thrombus. After GPNP-PA treatment, thrombus volume was reduced to approximately 75.1 % within 9 h. This enzyme-driven nanomotor can convert substrate biofuel into driving force, which greatly improves the efficiency of drug delivery.

#### 4.1.2 JNMs driven by multiple enzymes

In addition to single enzyme-driven JNM, triple enzyme-driven JNM has broader medical applications. Ma et al. fabricated self-propelled JNMs based on hollow mesoporous silica NPs (HMSNPs). This nanomotor was synthesized by using solid silica NPs as templates, modifying their surface amino groups to form HMSNP-NH_2_, and then depositing a 10 nm SiO_2_ layer onto the HMSNP-NH_2_ monolayer *via* electron beam evaporation to produce JHMSNP-NH_2_@SiO_2_. Finally, enzymes were attached to one side of the Janus NPs *via* glutaraldehyde linker molecules. The resulting nanomotors were powered by enzymatic reactions involving three distinct enzymes: catalase, urease, and glucose oxidase (GOx) (Fig. 6B) [26]. Propulsion of NPs by hydrogen generation through the reaction of metals with acid and water has the disadvantages of short lifetime and harsh reaction conditions [[105], [106], [107]]. In contrast, enzyme-driven JNMs have faster reaction rates and are used in a wider range of environments. To demonstrate that the nanomotor is driven by a biocatalytic chemical reaction, we analyzed the tracked nanomotor by means of a mean-square displacement analysis system *via* optical microscopy. We found a long time translational diffusion coefficient of 1.30 ± 0.05 μm^2^/s for JHMSNP-peroxidase compared to the value of 0.75 ± 0.03 μm^2^/s for no fuel, which is an increase of about 83 % at 1.5 wt% H_2_O_2_. And similar results were obtained by another technique, Dynamic Light Scattering, for measurements. The average diffusion coefficient of the nanomotor without fuel was 0.67 μm^2^/s, whereas this average diffusion coefficient increased by 85 % to 1.24 μm^2^/s at 1.5 wt% H_2_O_2_. In addition to catalase, the same was true for urease and catalase. After adding the corresponding fuels, the apparent diffusion coefficients of the two nanomotors increased by 52 % and 38 % under 25 mM urea and 500 mM glucose, respectively [108,109]. Therefore, the integration of nanomotors with enzymes would result in a novel enzyme-driven JNM, which is capable of performing a variety of functions in biological environments. Characteristics such as small size and hollow internal structure make these biocompatible enzyme-driven nanomotors have better prospects for development.

While enzymes provide power for JNM, they also cause drawbacks in JNM applications. As a biologically active substance, changes in pH, temperature, and microenvironmental components can affect enzyme activity, which in turn leads to a decrease in JNM performance [28,99,110]. In addition to enzyme inactivation, the cytotoxicity present in some enzymes and fuels needs to be considered urgently. Hydrogen peroxide-based JNM, which uses hydrogen peroxide as fuel, limits its application in physiological environments due to the cytotoxicity of hydrogen peroxide; glucose oxidase-based JNM produces toxic hydrogen peroxide, which is also cytotoxic. Moreover, enzymatic reactions may trigger biocompatibility issues, as exogenous enzymes can be recognized by the immune system as foreign substances, potentially inducing immune-mediated inflammation. Meanwhile, the enzyme-driven JNM system also exhibits substrate dependence. At excessively low concentrations, insufficient enzyme-substrate binding leads to inefficient system response, failing to meet functional requirements. Conversely, excessively high concentrations may trigger enzyme inhibition or non-specific side reactions, disrupting normal system operation. More critically, natural variations in substrate concentrations across different tissues and pathological sites within the body make precise regulation to system-compatible levels challenging, preventing stable performance in heterogeneous environments. In addition, most enzyme-driven JNMs rely on high concentrations of fuel for effective operation, so in practice, normal physiological environments may be difficult to meet such requirements [111].

### 4.2 Self-driven JNMs

Unlike the first four types of motions driven by applied conditions, the self-driven JNM realizes the property of motion by its own composition alone without the aid of any external force. It is based on the chemical reaction of the internal components themselves, and the reaction force generated by the gas produced by the reaction detaching from the JNM provides the impetus for the movement of the JNM. This self-driven method is free from the limitation of external force, which is much simpler, and the clinical application can greatly reduce the cost-effectiveness. Meanwhile, it has been widely used in biomedical fields, such as antimicrobial vascular synthesis and efficient blood coagulation, due to its self-driving ability and environmental adaptability. It has targeting, penetration, efficient release, and good biological adaptability, which can reach the tissue cells more accurately and improve the therapeutic effect, realizing efficient antimicrobial and vascular synthesis for lesions and efficient coagulation for bleeding sites.

**Fig. 7:**
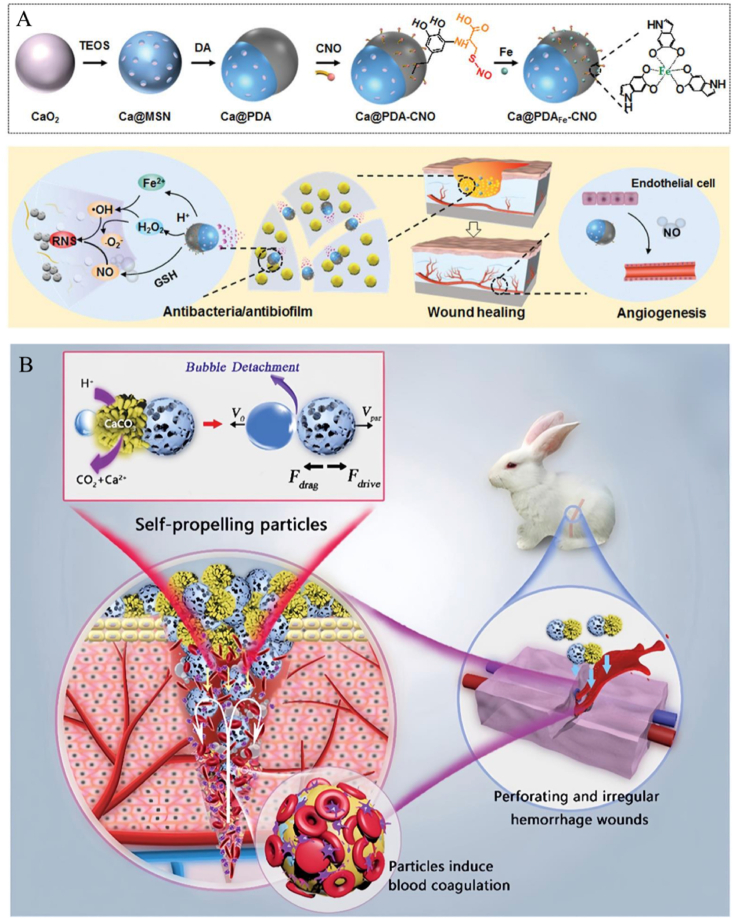
(A) Preparation process and working principle of Ca@PDAFe-CNO NPs. (B) Schematic representation of the synthesis process of Janus MSS@CaCO_3_T and its application. Reproduced with permission from Refs. [29,30].

In the treatment of diabetic foot ulcers, to address the presence of bacterial biofilm infections and impaired angiogenesis, a self-driven JNM with CaO_2_ NPs as the core, formation of a Janus structure by asymmetrically encapsulating PDA, and the subsequent introduction of Fe^2+^ and cysteine-NO modified JNMs (Ca@PDAFe-CNO NPs) (Fig. 7A) [29]. It triggers multistage NO release through the biofilm microenvironment for efficient antimicrobial and pro-angiogenic properties. CaO_2_ decomposes in an acidic biofilm microenvironment to produce O_2_ and H_2_O_2_, and O_2_ is released from one side of the Janus structure, driving the NPs to penetrate the biofilm. H_2_O_2_ reacts with Fe^2+^ *via* a Fenton reaction to produce -OH, and the high concentration of glutathione triggered the release of NO from CNO and further reacted with -OH to generate highly reactive nitrogen species, which significantly enhanced the bactericidal effect, while the released NO in turn promoted endothelial cell migration and angiogenesis. *In vitro* experiments showed that the NPs cleared more than 99 % of S. aureus biofilm and significantly accelerated wound healing and collagen regeneration in a diabetic mouse model. The self-driven nanomotor achieves dual functions of antibiotic-free antimicrobial therapy and pro-healing through environment-responsive drug release and autonomous motility, providing a new strategy for diabetic foot ulcer treatment.

In the clinical challenge of deep irregular bleeding that is difficult to stop, researchers have developed a self-driven Janus hemostatic particle (MSS@CaCO_3_T) that achieves efficient coagulation at deep bleeding sites by bubble drive (Fig. 7B) [30]. This NP is based on negatively charged modified microporous starch (MSS), which is modulated by surfactant and ethanol to grow flower-like CaCO_3_ crystals on the unilateral side of the MSS to form a Janus structure. Subsequently loaded with thrombin and protonated tranexamic acid (TXA-NH_3_^+^), the acid-base reaction of CaCO_3_ with TXA-NH_3_^+^ was utilized to generate CO_2_ bubbles to drive the particle movement. The MSS adsorbs blood components rapidly and concentrates coagulation factors through the microporous structure; Ca^2+^ and thrombin activate endogenous coagulation pathways and promote fibrin formation. Animal experiments showed that the particles stopped bleeding within 50 s and 3 min in liver and femoral artery hemorrhage models, respectively, and blood loss was reduced by more than 50 % compared with traditional hemostatic materials (*e.g.*, Celox). JNMs achieve deep bleeding targeting through bubble driving, combined with the adsorption capacity of microporous starch and the procoagulant effect of thrombin, significantly enhance the hemostatic efficiency of irregular bleeding, and have good biocompatibility and degradability. It also has good biocompatibility and degradability, providing an innovative solution for trauma first aid.

In solid tumor therapy, the autonomous intratumoral targeting and controlled release of drugs remain critical challenges. Conventional carriers face physical barriers within the tumor microenvironment and insufficient perfusion. To address these issues, Hou et al. designed a chemotactic anisotropic hollow multi-shell structure (a-HoMS) self-propelled nanomachine [118]. This system utilizes TiO_2_ as the substrate. By constructing an asymmetric double-shell hollow structure, the outer shell loads Au and Pt NPs to mimic glucose oxidase and catalase, respectively, forming a-HoMS-Au/Pt. This asymmetric structure establishes the foundation for chemotactic motion. Its self-propulsion relies on Au catalyzing the oxidation of tumor endogenous glucose into gluconic acid and H_2_O_2_, while Pt subsequently decomposes H_2_O_2_ to produce O_2_. This creates an asymmetric concentration gradient, driving the motor toward high-glucose regions *via* autodiffusion. Experiments demonstrate that this motor significantly enhances cellular uptake efficiency, achieves superior drug-induced apoptosis compared to traditional carriers, and exhibits no apparent toxicity.

The performance of a self-driven JNM may be significantly affected by factors in the surrounding environment, resulting in reduced stability. For example, when the self-driven JNM is applied in complex environments such as blood, the driving force generated by its bubbles may be interfered by factors such as blood composition and flow rate, resulting in decreased stability. Therefore, how to better improve the stability of the self-driven JNM still needs further research.

## 5 Composite-driven JNMs

Both externally field-driven JNMs and fuel-driven JNMs can be categorized as JNMs driven by single drive. While they exhibit significant performance improvements over traditional nanomotors, the inherent limitations of single-drive systems inevitably restrict their performance and application scope in complex environments. First, JNMs driven by a single drive are underpowered. For example, enzyme-driven JNM has limited catalytic sites and is largely dependent on substrate concentration, resulting in a weak propulsive force, which makes it difficult to effectively propel in complex biological fluids [119]. In addition, the single physical field drive leads to the problem of limited tissue penetration of JNM in complex environments, which will increase the difficulty of practical application. Secondly, the JNMs driven by a single drive have a single speed adjustment method, which makes it difficult to meet the needs of different environments and limits its application. In different application parts, the speed requirements are different, and a single speed regulation method cannot flexibly cope with diversified speed requirements. At the same time, in the face of different resistance interference, single drive JNM can not effectively adjust the speed to maintain movement, thus reducing work efficiency.

On the basis of a single drive force-driven JNM, researchers further proposed a composite-driven JNM. This composite-drive JNM has two or more propulsion modes to improve its motility in complex body fluids and extend its biomedical applications. Meanwhile, multiple composite drives can convert different energies into different motion mechanisms, which can better compensate for the drawbacks of a single drive, thus realizing complementary advantages as well as a strong combination (Table 5) [120].

**Table 5:** JNMs driven by composite driving forces.

Type	Composition	Size	Specific usage	Advantages	Remarks	Morphology	Schematic diagram	Ref.
Light-driven Enzyme-driven	SiO_2_@Au&PMO mPEG-SH GOx CAT	350 nm	Smart active drug delivery systems	1) Remote speed regulation 2) Excellent reproducibility 3) Effective in deep tissue	Asymmetric structure	–		[2]
Light-driven Ultrasound-driven	Ag-TiO_2_-LA	120 nm	Deep-seated infectious	1) High antibacterial efficiency 2) Enhanced penetration ability 3) Multimodal antibacterial effect	Synergistic SDT and PTT	Spherical		[128]
Light-driven Self-driven	TiO_2_/Au/Pt	–	Designing smart nanomachines	1) Dual-mode propulsion 2) On-the-fly motion control	Switchable propulsion modes	Spherical		[124]
	Core@Satellite Janus Mesoporous SilicaPt@Au	495 ± 20 nm	Intracellular drug delivery	Multi-phoretic propulsion	Switchable propulsion modes	Spherical		[125]
	DPSNs-NH_2_@Pt@CuS	–	Intelligent device applications	1) High loading capacity 2) Modulable motion speed	Asymmetric adsorption of NPs	Dendritic		[129]
Enzyme-driven Magnetically-driven	Au/MMP/Biotin-HPDP/BUs	2.21 ± 0.08 μm	Biosensing in microchips	1) High propulsion speed 2) Operable in high- viscosity fluids	1) Multilayered enzyme assembly 2) Enhanced catalytic sites	–		[119]
Magnetically-driven Ultrasound-driven	Au/Ni/Pt	Calibr: 400 nm Length: 3 μm	Nanoscale manipulation and assembly	1) Operable in complex medi 2) Controllable collective behavior	Rapid speed and direction control	Rod-like		[126]
Light-driven Enzyme-driven Self-driven	Janus DMS/C@Pt	986 nm	Biomedical applications	1) High enzyme loading capacity 2) Potential in treating triglyceride-related diseases	Dendritic porous structure for enzyme loading	Snowman-shaped		[120]

**Fig. 8:**
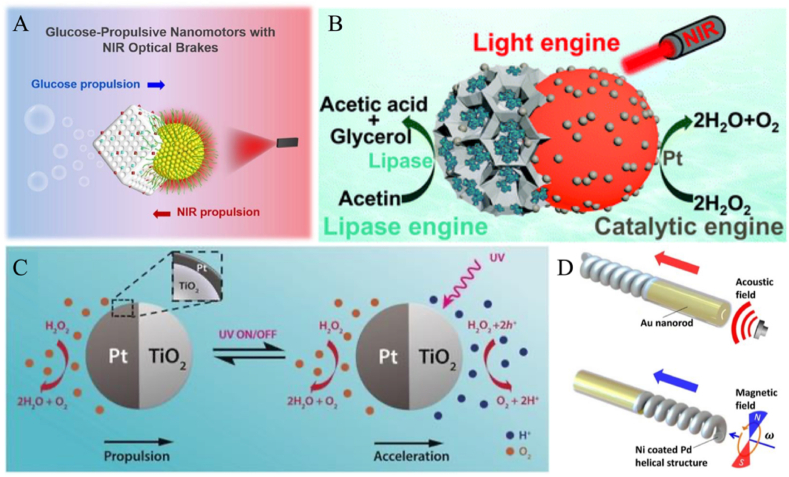
(A) Schematic mechanism of motion of SiO_2_@Au& PMO-enzyme nanocomposites. (B) Principle of motion of LDMS/C@Pt. (C) Propulsion mechanism of TiO_2_/Pt JNMs. (D) Schematic design of magnetoacoustic composite-driven JNMs and its propulsion mechanism. Reproduced with permission from Refs. [120,124]. Reprinted with permission from Ref. [2]. Copyright 2022 American Chemical Society. Reprinted with permission from Ref. [31]. Copyright 2015 American Chemical Society.

As a dual-powered JNM, the enzyme- and light-co-driven JNM utilizes the catalytic reaction of enzymes and the light response to synergistically drive the NP motion. As one of the most typical chemical fuel-driven nanomotors, the enzyme-driven nanomotor relies on the efficient biocatalytic reaction of biofuel inherent in the natural biological host, which can generate a powerful driving force for efficient propulsion [[121], [122], [123]]. However, speed as one of the main parameters of JNM motion, precise speed regulation of enzyme-driven JNM is very important in its biological applications. Inspired by automobile brakes, Liu et al. introduced NIR as an “optical brake” into enzyme-driven JNMs, combining optical and enzymatic drives to realize remote speed regulation for the first time (Fig. 8A) [2]. They designed asymmetric mesostructured SiO_2_@Au&organic silica-enzyme nanocomposites with Au nano-shells modified with GOx and catalase on periodic mesoporous organic silica. In a glucose solution, the nanomotor is driven exclusively by the enzyme on one of the sides, whereas if NIR radiation is added, the Au side generates a drive in the opposite direction, which results in speed regulation. In *in vitro* experiments, to assess the remote controllability of the nanomotor in biological tissues, the researchers placed 2 mm of pork muscle tissue between the NIR light and the JNM. The directional motion (blue line) of this JNM was clearly visible in a 100 nm glucose solution, while the directional motion (red line) was significantly suppressed after irradiation with NIR.

While speed is important in complex and variable body fluid environments, it is also a great challenge to ensure the efficient movement of JNMs for a long period of time. Based on the combination of enzymatic and optical dual propulsion, Xing et al. reported a lipase-modified dendritic silica/carbon@Pt (LDMS/C@Pt) JNM (Fig. 8B) [68]. One side of the material consists of SiO_2_@Au core-shell nanospheres, while the other side comprises periodic mesoporous organosilicon (PMO) nanocubes modified with glucose oxidase (GOx) and catalase (CAT). The prominent photothermal properties of the carbon material enable LDMS/C@Pt to achieve thermoelectrophoretic propulsion under NIR light irradiation, and the lipase loaded in the dendritic pores enables triacetate-driven motions, in addition to the asymmetrically distributed Pt NPs in LDMS/C@Pt itself, which catalyzes the decomposition of H_2_O_2_ and propels the motor through an oxygen concentration gradient. It was demonstrated that the motion trajectory of DMS/C@Pt was lengthened with the increase of H_2_O_2_ solution concentration; the motion of DMS/C@Pt was enhanced with the increase of near-red light power density; and the LDMS/C@Pt propulsion was enhanced with the increase of substrate concentration. In this way, due to the different components, it can be used to flexibly customize different driving modes, so that the collection of the advantages of enzyme-driven, light-driven, and self-driven can help to overcome the problem of limited fuel concentration and improve the possibility of effective propulsion.

Currently, research on JNMs combining self-driven and light-driven modes is also gaining significant traction [124,125]. Among them, Chen et al. designed a TiO_2_/Au/Pt JNM, a dual-mode JNM, which can realize “dynamic” motion control by adjusting the chemical reaction on the surface of the micromotor in real time (Fig. 8C) [124]. Pt catalyzes the decomposition of H_2_O_2_, and TiO_2_ photocatalyzes under UV light to counteract the chemical propulsion generated on the Pt side. To realize the motion control. Experimental 2D simulations show the distribution of released protons around the surface of the TiO_2_/Au/Pt JNM at different fuel concentrations and light intensities. In a 10 % H_2_O_2_ solution and under no light conditions, protons accumulate near the surface of Pt, resulting in the propulsion force pointing from TiO_2_ to Pt. Whereas under UV irradiation (1 W/cm^2^), photogenerated holes on the surface of TiO_2_ oxidize the H_2_O_2_ to produce protons, resulting in a symmetric distribution of protons around the micromotor so that the two driving forces are in equilibrium and the motion stops in this case. However, when the concentration of hydrogen peroxide decreases to 2.5 %, the catalytic reaction on the Pt surface is inhibited, so the photoelectrochemical reaction on the TiO_2_ surface becomes the dominant reaction (1 W/cm^2^), the protons accumulate on the TiO_2_ side, and the direction of the driving force changes. In this way, the TiO_2_/Au/Pt JNM realizes dynamic motion control, and the motion trajectory of the TiO_2_/Au/Pt JNM in the experimental video has visual proof.

In addition to the aforementioned composite-driven JNMs, magnetically driven acoustically driven composite-driven JNMs and magnetically driven enzymatically driven composite-driven JNMs also exhibit high efficiency. The magnetoacoustic composite-driven JNMs developed by Li's team can be driven by either magnetic or ultrasonic fields, which can be used in a wide range of imaging, diagnostic, and drug delivery applications [126]. In addition to the application of magnetism in imaging, Luo et al. used magnetism to realize the orientation regulation of JNMs (Fig. 8D) [31]. They immobilized multilayers of biotinylated urease in an asymmetric distribution in biotinylated Janus Au/magnetic particles with the assistance of streptavidin. This JNM, which significantly increased the amount of urease, could reach an average self-driven speed of about 21.5 ± 0.8 μm/s at physiological urea concentration (10 mM), which is six times higher than that of the single-layer micro-motor. In addition, the inherent magnetic properties of this JNM enable it to realize the modulation of motion direction under the action of an applied magnetic field.

In the medical field, chemokinesis therapy (CDT) is widely used for tumor treatment. However, current nanomaterials employed in CDT struggle to effectively penetrate tumors due to their lack of motility, resulting in suboptimal therapeutic outcomes. To address this, Xing et al. developed bio-inspired zebrafish carbon/manganese nano-engines (JCMNs), which also exhibit an asymmetrical structure mimicking jellyfish morphology. These engines achieve enhanced CDT treatment through dual propulsion using H_2_O_2_ and NIR light [127]. Distinctively, its core comprises a carbon-based skeleton with irregular surface protrusions. Carbon components are uniformly distributed throughout the core and protrusions, while manganese components (MnO_2_ nanosheets) are generated *via* redox reactions with the carbon skeleton and preferentially loaded onto the surface of the “bell-shaped” core. The balanced ratio of carbon and manganese components ensures both H_2_O_2_ catalytic activity and sufficient carbon content to maintain NIR photothermal conversion efficiency, thereby enhancing energy consumption. Intracellular motility experiments demonstrate that JCMNs can initiate propulsion in intracellular environments with H_2_O_2_ concentrations below 100 μM, exhibiting significantly extended trajectories and broader coverage, proving their dynamic mobility within cellular microenvironments.

To summarize, the composite-driven JNM is based on the single drive JNM, combining the advantages of two and more propulsive forces to make up for the shortcomings of a single propulsive force, such as environmental dependence problems, stability problems, and so on. Below, we compare the performance metrics of single-drive and composite-drive JNMs (Table 6). We anticipate the development of more composite-driven systems JNMs, which will better compensate for the limitations of single-drive modes and enable broader applications in biomedicine.

**Table 6:** Comparison between single drive system and dual drive system.

Shape of JNMs	Speed (μm/s)	Effective load capacity	Energy efficiency	Ref.
Light-driven JNMs	15.2 ± 1.0	Middle, it can carry photothermal agents (gold, Au) and drugs (paclitaxel, PTX) with a drug loading capacity of 8–12 %.	The efficiency of near-infrared photothermal conversion is 30–50 %; But it is affected by the absorption and scattering of biological tissues	[30,78]
Enzyme-driven JNMs	12.1 ± 0.9	High, thrombolytic drugs (urokinase) and chemotherapy drugs (doxorubicin, DOX) have a loading capacity 30 % higher than ordinary nanoparticles.	Relying on the catalytic mechanism of biofuels, it has strong adaptability to substrates such as glucose and urea *in vivo*; Catalytic efficiency is 25 % higher than that of free enzymes	[16,99]
Magnetically-driven JNMs	18.3 ± 1.3	Middle, such as regorafenib + doxorubicin, the loading amount is about 10–15 %	The energy conversion rate of the magnetic field is stable at 60 %–70 %, and there is almost no energy loss or chemical fuel consumption during the energy transfer process	[83,114]
Ultrasound-driven JNMs	32.4 ± 1.8	Middle, it can be loaded with thrombolytic drugs (urokinase) and photosensitizers (indocyanine green, ICG), with a drug loading of 12–18 %.	The energy conversion rate of ultrasonic cavitation is 25 %–40 %	[23]
Light-driven Enzyme-driven	35.0 ± 2.3	High, capable of carrying gold (Au) photothermal agent, glucose oxidase (GOx), and anti proliferative drugs (paclitaxel, PTX), with a drug loading 60 % higher than single light driven JNMs, reaching 18–22 %	The near-infrared photothermal conversion efficiency has been increased to 50 %, while the enzyme catalytic efficiency is 30 % higher than that of single enzyme drive	[2,121]
Light-driven Ultrasound-driven	41.8 ± 2.4	Middle, it can carry silver (Ag) photothermal agent + titanium dioxide (TiO _2_) + antibiotics, with a drug loading 45 % higher than single ultrasound driven JNMs, reaching 17–22 %	The near-infrared photothermal conversion efficiency is 40 %, the ultrasonic cavitation efficiency is 35 %, and the total energy efficiency is 0.8 times higher than that of single ultrasonic driven JNMs	[130]
Enzyme-driven Magnetically-driven	21.3 ± 1.3	High, can carry multi-layer urease + chemotherapy drug (doxorubicin) + targeted ligand (anti-VCAM-1 antibody), drug loading is 50 % higher than single enzyme driven JNMs, reaching 20–25 %	By assembling multiple layers of enzymes, the catalytic efficiency is increased by 40 %, combined with a magnetic field orientation efficiency of 90 %. The total energy efficiency is twice as high as that of single enzyme driven JNMs	[31]
Magnetically-driven Ultrasound-driven	54.8 ± 2.7	Middle, it can carry magnetic particles of iron trioxide (Fe ∝ O _4_) and ultrasound responsive drugs (urokinase), with a drug loading capacity 30 % higher than single magnetic driven JNMs, reaching 13–18 %		[126]

## 6 Clinical translation challenges

JNMs face challenges in toxicity, production, *in vivo* behavior, and regulation as they transition from the laboratory to clinical practice.

In terms of toxicity and biological safety, some nanomotors have complex problems due to the presence of metal components. At high concentrations, metal particles readily agglomerate, and excessive phagocytosis by immune cells may trigger cellular-level damage such as lysosomal injury. Unmodified metal surfaces readily bind non-specifically to blood components, triggering immune responses. Moreover, most metals are non-degradable, and their long-term accumulation in the body can increase the metabolic burden on organs like the liver and kidneys. Certain metals, if at risk of ion release, may also interfere with normal cellular physiological functions. Additionally, improper particle size or surface modification of metal particles could allow them to penetrate biological barriers, potentially causing damage to healthy tissues.

At the production level, there is currently a lack of unified clinical-grade quality standards. The detection technologies for key indicators such as the motion performance of nano-motors and drug loading stability at the nanoscale are still not perfect, making it difficult to achieve precise quality control throughout the entire process. More critically, the multifunctional integration required for clinical applications—such as actuation, targeting, and imaging—significantly increases synthesis complexity. Functional interference often arises during multicomponent coordination, making it extremely challenging to maintain the stability and synergy of all functions during large-scale production.

The collective motion of nanomotors offers a new approach to addressing the aforementioned challenges in clinical applications. The collective motion of JNMs is a dynamic process wherein multiple JNMs form an ordered collective through synergistic interaction to achieve synchronized movement. Its core value lies in overcoming the limitations of single motors in complex physiological environments, such as weak propulsion, susceptibility to biofluid interference, and difficulty penetrating deep tissues. Leveraging their “swarm collaboration” properties, JNMs significantly enhance physiological barrier penetration efficiency and targeted delivery capabilities, making them a key direction for translating basic research into clinical applications in this field. The orderly regulation of collective motion relies primarily on the synergistic interaction of two pathways: “external field control” and “microenvironment response.” In external field control, magnetic actuation and NIR light regulation are common strategies. The former guides the motor to dynamically reconfigure its cluster morphology to adapt to different physiological environments, while the latter induces synergistic effects within the cluster to enhance penetration capabilities. Microenvironment response strategies leverage specific biochemical gradients at pathological sites like tumors to drive clusters toward lesion cores while reducing adhesion resistance against biological barriers. In applications targeting physiological barriers, collective motion demonstrates exceptional advantages. It can address multiple scenarios—including the blood-brain barrier, alveolar-capillary barrier, and tumor stroma barrier—by synergistically softening barrier structures, overcoming mucus obstacles, and optimizing drug distribution. However, clinical translation still requires addressing biosafety and movement precision challenges. Future progress will depend on multidimensional integration—combining biodegradable material design with intelligent algorithmic control—to advance MNMs toward precision therapy.

Therefore, to achieve clinical transformation, it is necessary to develop degradable metal materials, innovate the preparation methods of JNMs, and promote the formulation of dedicated regulatory guidelines.

In the past two years, JNMs has also achieved multi-dimensional breakthroughs in precise targeted delivery and clinical translation. In gene editing, Shen et al. reported ROS-driven RDN@PL JNMs. These devices encapsulate heme-core CRISPR/Cas9 plasmids targeting LDHA, leveraging tumor ROS gradients to penetrate solid tumors and achieve a 93.9 % growth inhibition rate [131]. In adaptive drug release, AI-assisted design has emerged as a novel approach. Gui et al. optimized the drug release kinetics of pH/GSH dual-sensing JNMs by simulating tumor microenvironment response mechanisms *via* AI, increasing chemotherapy drug utilization by 40 % [132]. Magnetic JNMs accelerate clinical translation. Sun et al.'s Magneto-JNMs combined with MRI targeting achieved 85 % tumor-targeted enrichment in Phase I trials for advanced liver cancer patients [133]. These breakthroughs propel JNMs from fundamental research toward diversified clinical applications.

The long-term development of JNMs requires a balance between ethical safety, environmental sustainability, and industrial feasibility. On an ethical level, it is necessary to adopt biodegradable Fe_3_O_4_、PLA、Microporous starch and other materials are used to replace traditional non degradable materials such as Au, SiO_2_, PS, *etc.,* thereby promoting the greening of the preparation process. A waste JNMs recycling system and life cycle assessment (LCA) standards are established to reduce environmental risks. At the same time, long-term animal experiments are conducted to monitor the toxicity of degradable materials *in vivo*, following clinical ethical norms such as informed consent and animal experiment 3R principles to ensure biological safety; At the industrial level, we aim to reduce material, equipment, process, and testing costs through material substitution, equipment upgrades, process optimization, and integrated online testing. Our goal is to keep mass production costs below $50/g to maintain market competitiveness. At the same time, we focus on core patent layouts for preparation and driving technologies, and address patent risks through cross licensing, filling sustainable material patent gaps, and international patent layouts; Commercialization can be promoted in three stages: short-term (1–3 years, completing preclinical validation and pilot testing, applying for core patents, targeting trauma first aid), medium-term (3–5 years, achieving large-scale production, conducting clinical trials, targeting cancer and trauma treatment), and long-term (5–10 years, expanding multi disease applications and establishing industry standards, promoting global market expansion). In the future, ethical safety and industrial feasibility need to be included in the early design stage of JNMs to promote their transition from technological innovation to clinically available and market accessible nanotherapy tools [134].

## 7 Conclusion and perspective

JNMs rely on the active movement ability and multifunctional integration characteristics of asymmetric structures to form targeted application directions in the treatment of various diseases. In the treatment of malignant tumors, light-driven JNMs can break through the tumor interstitial barrier and enhance drug enrichment, magnetically-driven JNMs are suitable for precise targeting of deep tumors, and enzyme or self driven JNMs utilize endogenous fuels in the tumor microenvironment to achieve autonomous movement. Multiple types of drivers improve efficacy from the dimensions of penetration, targeting, and environmental adaptation. For ischemic diseases, ultrasound-driven JNMs can open the blood-brain barrier to deliver neuroprotective drugs, while light-driven JNMs regulate the release of angiogenic factors to improve limb ischemia. Magnetically-driven JNMs target and accumulate in myocardial ischemic areas to reduce inflammation and fibrosis, addressing physiological barriers and drug delivery issues in different ischemic scenarios. In the treatment of infectious diseases, enzyme-driven JNMs can degrade the bacterial biofilm structure. Light-driven JNMs combine the photothermal and photocatalytic effects to kill drug-resistant bacteria. Self-driven JNMs can penetrate the biofilm at the infected site and simultaneously promote tissue repair, effectively dealing with biofilm obstruction and drug-resistant bacterial infection. For thrombotic diseases, light-driven JNMs use photothermal effects to degrade thrombotic components. Ultrasound-driven JNMs can respond to the thrombotic microenvironment for synergistic thrombolysis and endothelial repair, overcoming the limitations of low efficiency and high recurrence risk in traditional thrombolysis. These applications are based on precise matching of different driving mechanisms of JNMs with disease treatment needs, providing innovative nanotools for efficient treatment of multiple diseases.

However, even though JNMs driven by different driving forces have great advantages, their problems cannot be ignored. At the application level, the controllable fabrication of JNMs involves complex processes such as multi-step self-assembly and template synthesis. These not only demand highly specialized equipment and skilled operations but also result in lengthy preparation cycles and significant material wastage. Consequently, production costs remain prohibitively high, severely hindering industrial-scale manufacturing and commercialization. In practical scenarios, JNM suffers from multiple interferences in complex chemical environments, such as pH fluctuations, changes in ionic strength, and degradation by proteases and reactive oxygen species within biological systems. These factors accelerate structural damage and functional component failure, significantly reducing their motion performance, targeting capabilities, and payload release efficiency. Furthermore, existing drive systems have inherent limitations. Light-driven JNM is limited by light penetration depth and tissue scattering effects, making deep tissue applications challenging. Enzyme-driven JNM suffers from unstable motility due to substrate concentration fluctuations, enzyme activity decay, or competitive inhibition. While composite-driven JNM partially integrates multiple drive advantages, it still suffers from issues such as low therapeutic component release efficiency and insufficient imaging contrast, making it difficult to meet the stringent standards for clinical translation.

Although there are multiple limitations in the current JNM preparation, the current development of certain technologies provides ideas for the large-scale and low-cost production of JNM. Among them, 3D printing technology will show great potential in the processing of JNMs. Through high-precision 3D printing, the customization of complex structures can be achieved, and the precise control of the geometry, size and internal structure of JNMs will help to better optimize their kinematic properties. In addition, cutting-edge technologies such as nanoimprinting and photolithography can also be used for the fine processing of JNMs to prepare JNMs with special structures and functions. In addition to the five types of single-driver-driven JNMs and different combinations of composite-driven JNMs mentioned in this paper, there are many other drivers that have not yet been fully utilized. For example, motion is achieved by relying on electrical charges generated by friction between materials. In addition, biomimicry-driven motion mechanisms can also provide ideas for motion innovations in JNMs. For example, we can refer to the swimming mode of sperm to realize more flexible motion regulation and speed regulation. Through the improvement of the production technology and the novel driving force of movement, it is expected to develop a superior JNM and significantly enhance its application in complex environments.

Looking ahead, JNMs driven by different driving forces are expected to make significant breakthroughs in several fields. Breakthroughs in NP fabrication and smart materials technology will dramatically reduce the manufacturing cost of JNMs and drive the clinical translation process. This will not only realize a controllable intelligent drug delivery system, but also build an integrated platform for diagnosis and treatment, which will fundamentally realize the intervention of diseases. The current motion control of JNM faces problems of environmental dependence, insufficient coordination, and poor stability, while intelligent response faces problems of single stimulus, no feedback, and functional disconnection. In the future, biological adaptation should be the core, breaking through technological bottlenecks through new driving mechanisms, dynamic feedback systems, and functional collaborative design, while promoting material innovation and large-scale preparation, ultimately achieving the transformation from laboratory controllability to clinical usability. At the level of application frontierization, JNMs open up a new dimension of medical treatment. In the future, they can perform microscopic operations and overcome the blind spots of rare disease treatment. We believe that the socio-economic impact of JNMs will be gradually expanded, and the improvement of the efficiency of targeted therapy can shorten the treatment course and reduce the recurrence rate of the disease. The step-by-step improvement and development of JNMs will promote the progress of the nanomedicine era and bring more benefits to human health.

## CRediT authorship contribution statement

**Banghui Wang:** Writing – original draft, Visualization, Software, Resources, Methodology, Investigation, Formal analysis, Data curation, Conceptualization. **Tao Chen:** Writing – original draft, Visualization, Supervision, Software, Resources, Project administration, Investigation, Data curation, Conceptualization. **Yixuan Li:** Writing – original draft, Visualization, Validation, Supervision, Software, Data curation, Conceptualization. **Tong Yin:** Writing – original draft, Software, Resources, Formal analysis, Data curation. **Zeyu Xi:** Writing – original draft, Software, Resources, Funding acquisition, Formal analysis. **Yuhan Guo:** Writing – original draft, Resources, Data curation, Conceptualization. **Yuanhong Xu:** Writing – review & editing, Visualization, Validation, Investigation, Funding acquisition, Data curation. **Xian-Ming Chu:** Writing – review & editing, Validation, Supervision, Methodology, Investigation, Funding acquisition, Formal analysis.

## Declaration of competing interest

The authors declare that they have no known competing financial interests or personal relationships that could have appeared to influence the work reported in this paper.

## Data Availability

Data will be made available on request.
